# Activity against *Mycobacterium tuberculosis* of a new class of spirooxindolopyrrolidine embedded chromanone hybrid heterocycles†

**DOI:** 10.1039/d4ra01501k

**Published:** 2024-04-11

**Authors:** Manal Fahad Alkaltham, Abdulrahman I. Almansour, Natarajan Arumugam, Siva Krishna Vagolu, Tone Tønjum, Shatha Ibrahim Alaqeel, Saiswaroop Rajaratnam, Venketesh Sivaramakrishnan

**Affiliations:** a Department of Chemistry, College of Science, King Saud University P.O. Box 2455 Riyadh 11451 Saudi Arabia anatarajan@ksu.edu.sa; b Department of Microbiology, University of Oslo N-0316 Oslo Norway vagolu000@gmail.com; c Department of Microbiology, Oslo University Hospital N-0424 Oslo Norway; d Department of Chemistry, College of Science, King Saud University (034) Riyadh 11495 Saudi Arabia; e Disease Biology Lab, Department of Biosciences, Sri Sathya Sai Institute of Higher Learning Prasanthi Nilayam Anantapur Andhra Pradesh India

## Abstract

A new class of structurally intriguing heterocycles embedded with spiropyrrolidine, oxindole and chromanones was prepared by regio- and stereoselectively in quantitative yields using an intermolecular tandem cycloaddition protocol. The compounds synthesized were assayed for their anti-mycobacterial activity against *Mycobacterium tuberculosis* (*Mtb*) H37Rv and isoniazid-resistant (*katG* and *inhA* promoter mutations) clinical *Mtb* isolates. Four compounds exhibited significant antimycobacterial activity against *Mtb* strains tested. In particular, a compound possessing a fluorine substituted derivative displayed potent activity at 0.39 μg mL^−1^ against H37Rv, while it showed 0.09 μg mL^−1^ and 0.19 μg mL^−1^ activity against *inhA* promoter and *katG* mutation isolates, respectively. A molecular docking study was conducted with the potent compound, which showed results that were consistent with the *in vitro* experiments.

## Abstract 2

Spirooxindolopyrrolidines were synthesized in quantitative yield through cycloaddition strategy. Compounds exhibited significant anti-tubercular activity and molecular docking studies of the compound is well correlates with *in vitro* findings.

## Graphical abstract

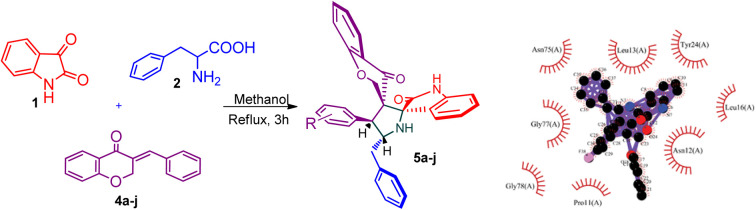

## 1. Introduction

Tuberculosis (TB) is one of the deadliest airborne infectious diseases, caused by various species and strains of *mycobacteria*. Among them, pathogenic aerobic bacteria, the *bacillus Mycobacterium tuberculosis*, remains the major cause of TB in humans. *Mycobacterium tuberculosis* usually forms an infection in the lungs of the host and is one of the leading unfavorable health problems globally. World Health Organization report in 2022 indicate that TB has replaced COVID-19 as the leading cause of death from an infectious agent on a worldwide basis.^1^ It is estimated that 85% of drug-susceptible TB cases can be cured with the current therapeutic treatment.^2^ Several first-line anti-TB medications are available, including rifampicin, isoniazid (INH), pyrazinamide, and ethambutol. These drugs have associated side effects, are ineffective in eliminating latent pathogens, and require prolonged treatment.^3^ Second-line drugs like bedaquiline, delamanid, or pretomanid are less effective, has a higher toxicity profile, and is more expensive than the first-line medication. Moreover, the current evolution of multidrug-resistant TB further aggravates the complications associated with TB treatment. Thus, the disease continues to be a major social health issue, particularly among low-income populations.^4,5^ TB in combination with HIV^5^ increases the overall incidence of TB by 50 times more in HIV-positive patients than HIV-negative individual.^6^ Therefore, it is necessary to develop structurally novel, potent, fast acting, affordable anti-TB drugs with an innovative mechanism of action and low toxicity profile that are capable of overcoming the mechanisms of resistance posed by existence of multidrug and extensively drug resistant tuberculosis (MDR-TB and XDR-TB) in order to effectively combat TB.

In this context, nitrogen and oxygen comprising heterocyclic hybrid frameworks play crucial contribution in drug discovery, a significant part of which is the fact that they are the active entrants in a many number of drugs.^7^ In addition, molecules embedding heteroatoms possess promising drug solubility and pharmacokinetic properties. It has been argued that recent trends in the discovery of lead compounds have led to a “escape from flatland” in which planar aromatic or heteroaromatic ring systems are a rapidly replacing others with higher saturation and three-dimensionality. Such molecules are anticipated to have a better affinity for three-dimensional binding pocket in proteins and be more solubilized which is an essential property for drug development and practical applications.^8,9^

In this connection, spiro compounds have emerged as an attractive synthetic target in drug discovery,^10–15^ due to their structural complexity and rigidity, facility to reveal functionality and inherent three-dimensional structural features, which offer improved structural affinity to biological targets. Perhaps for this reason, many spiro compounds are found in biologically active alkaloids and synthetic products that have evolved to interact with target proteins of the biological system^15^ more readily than flat hetero aromatic ring systems. In this context, R. R. kumar *et. al*.^16^ reported an atom economic, stereoselective synthesis and antimycobacterial evaluation of spiropiperidine through cycloaddition methodology. Among these spiropiperidines, fluoro substituted compounds showed excellent activity against tested TB organism. Similarly, a library of spirooxindolopyrrolidines/pyrrolizidines/pyrrolothiazole derivatives were synthesized and assayed for their antimycobacterium tubercular activity by S. M. Rajesh *et. al*.^17^ and found that most them demonstrated excellent activity against tested TB-organism. Recently, M. Ganesh *et. al.*^18^ reported the synthesis, characterization, molecular docking simulation and antitubercular activity of new class of spiropyrrolidine oxindoles, these compounds showed significant activity against tested *Mycobacterium tuberculosis* H37R. In addition, to the biological precedents of the spiro compounds described above, most of the spiropyrrolidines reported in the literature demonstrated significant biological activity, including anti-TB activity,^19,20^ some of these spiro compounds were even more potent than reference standard.^21–24^ Prompted by these finding, herein we report the synthesis and anti-tubercular activity of structurally diverse spirooxindolopyrrolidine integrated chromanones using three component reactions involving 1,3-dipolar cycloaddition reaction.^25–29^

## 2. Results and discussion

### 2.1. Chemistry

**Scheme 1:**
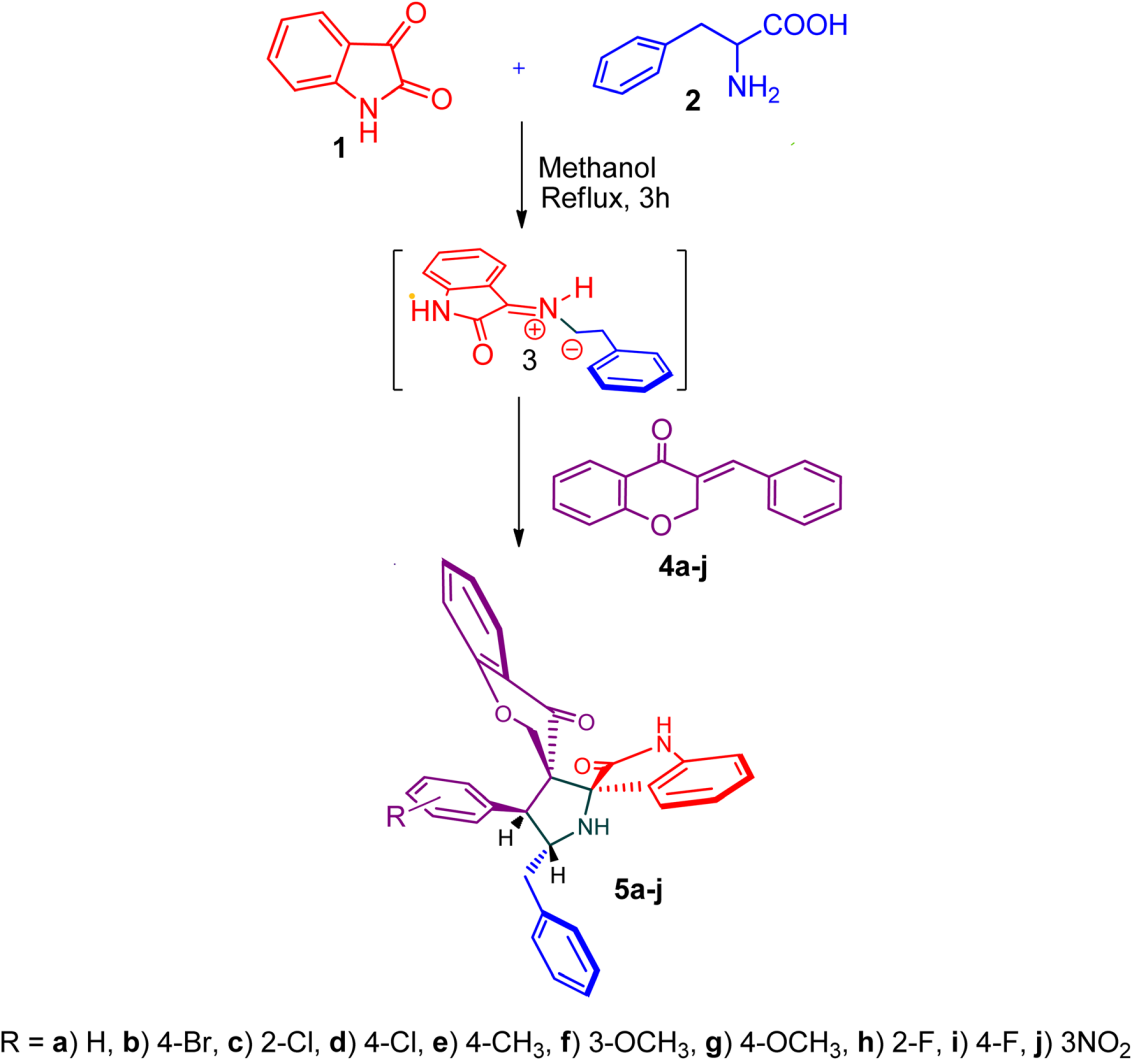
Synthesis of dispiropyrrolidine tethered chromanone hybrids, 5a–j.

**Fig. 1:**
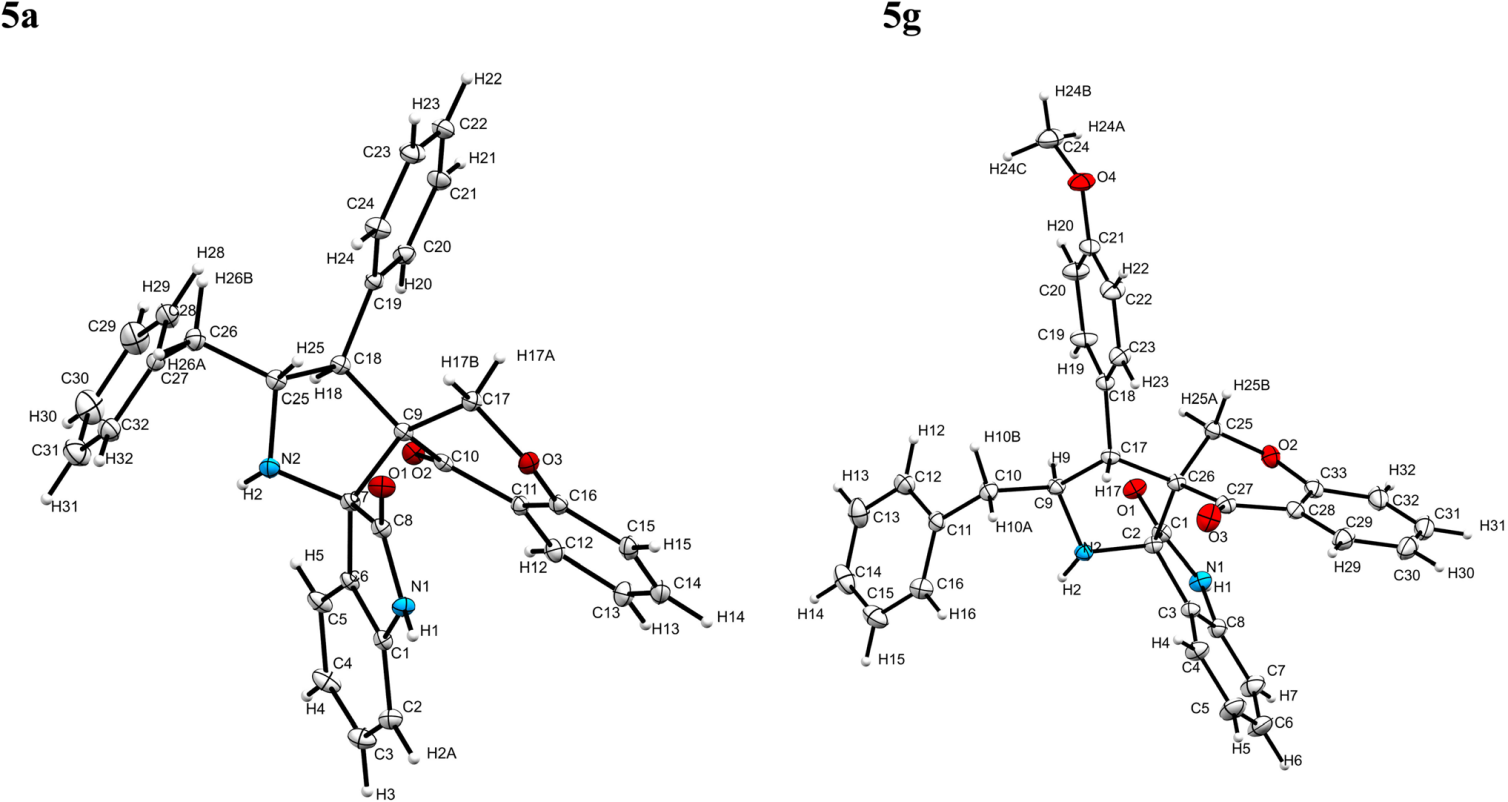
Crystal structure of compounds 5a and 5g.

The cycloaddition protocol initiated by the reaction of 3-arylidenechroman-4-ones 4 and *in situ* 1,3-dipole prepared from diketone 1 and amino acid 2 under reflux in MeOH yielded exclusively the substituted spiropyrrolidine heterocycles in quantitative yields (88–95%, Table 1) as described in Scheme 1. In the initial stage of optimization, several solvent systems were used including EtOH, MeOH, CH_3_CN, DMF and CH_3_CN : MeOH (1 : 1 v/v). An excellent yield was obtained in MeOH than to other solvents, indicating that MeOH is suitable for the reaction. Thus, the mixture of arylidene chromanone 4, diketone 1, and amino acid 2 is refluxed in MeOH until the reaction is completed, as indicated by TLC and the products 5a–j are purified by crystallization. The spectroscopic analysis unambiguously assigned by the structure of compounds (*vide*, ESI†). Finally, X-ray diffraction analysis described in Fig. 1 confirmed the stereochemistry and regio of the structures 5a and 5g.^30^

**Table 1:** Spiropyrrolidine tethered chromanone heterocyclic hybrids, 5a–j

Entry	Structure of compounds	Melting point (°C)	Yield^a^ (%)
1	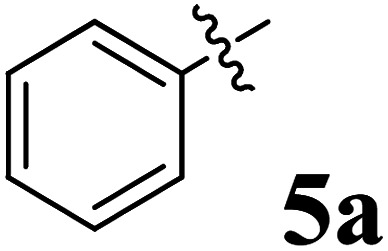	188–190	89
2	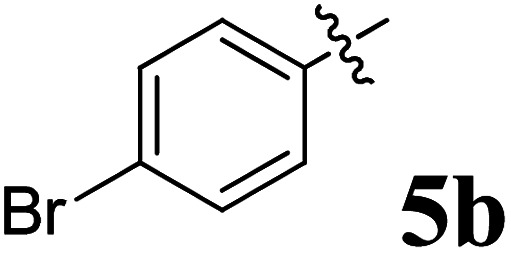	186–188	94
3	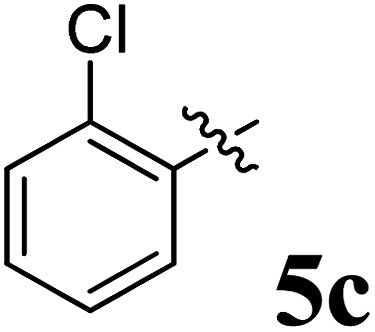	182–184	92
4	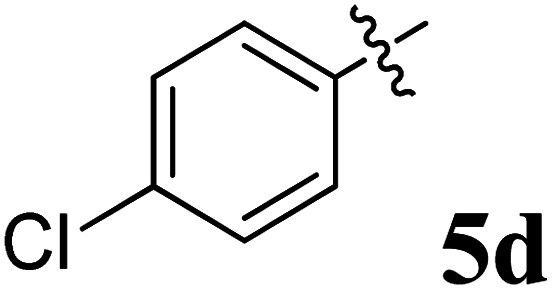	151–153	96
5	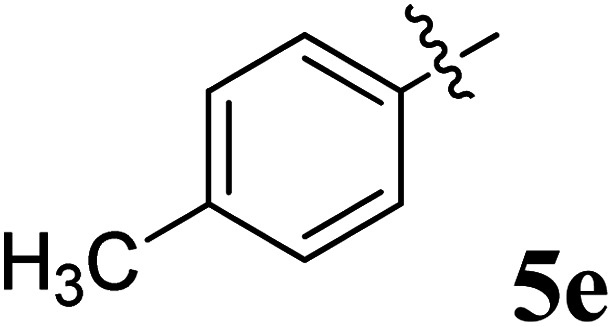	153–155	87
6	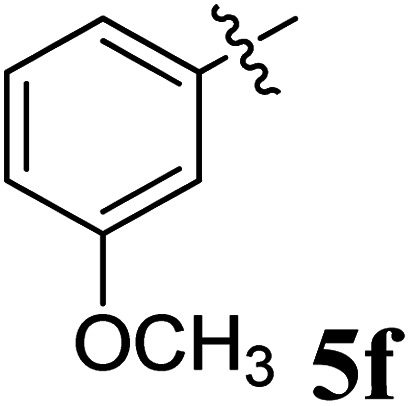	176–178	85
7	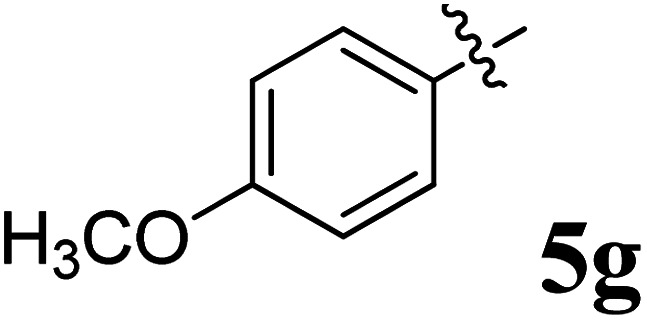	185–187	88
8	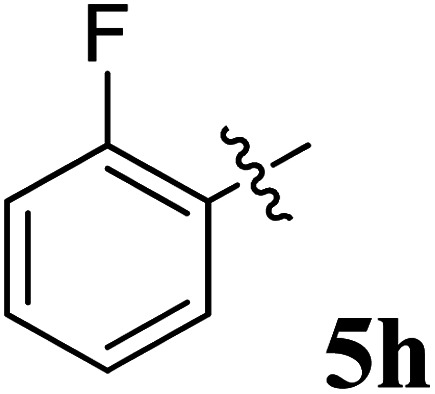	152–154	90
9	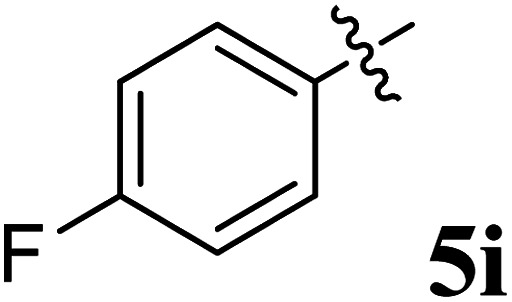	152–154	92
10	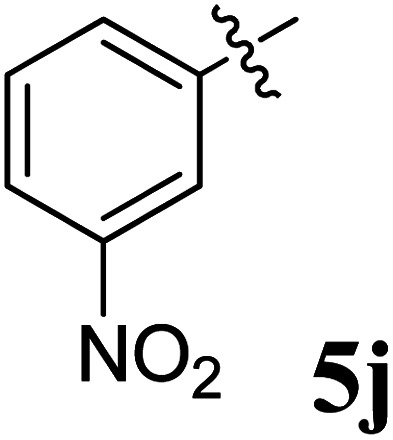	163–165	91

a Isolated yield of cycloadduct.

**Scheme 2:**
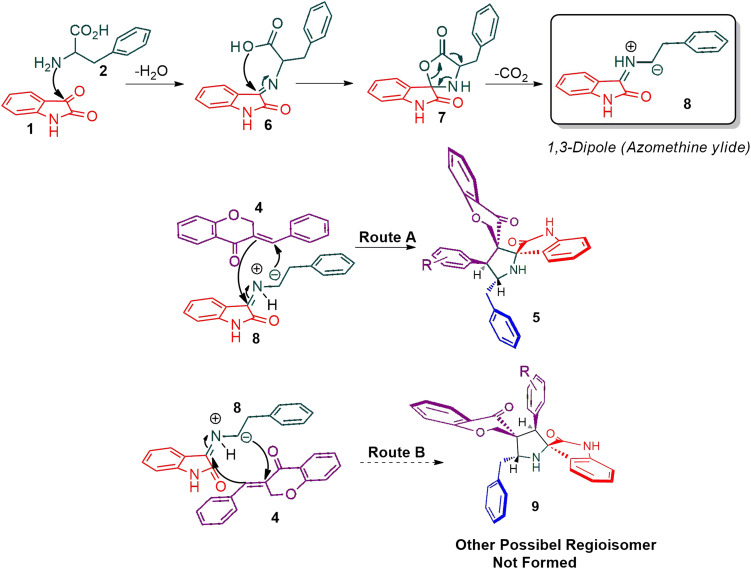
The persuasive pathway for the construction pyrrolidine, 5.

Scheme 2 shows a possible mechanism for the construction of spiropyrrolidine 5. In the first step, the reaction of active ketone 1 and l-phenylalanine 2 produced the non-stabilized azomethine ylide 8*via* intermediates 6 and 7 under spontaneous decarboxylation reaction process. The non-stabilized ylide 8 was further reacts with benzylidene chromanone 4 yielded sole cycloadduct 5*via* route A. Furthermore, the reaction proceeded regioselectively, as no traces of the possible regioisomer 9 were detected. The dipole 8 adds to the electron less carbon of the chromanone 4, producing cycloadduct 5. Four new stereogenic carbons, including two spirocarbons, were formed in this reaction process, resulting from the construction of two C–C and one C–N bonds.

### 2.2. Anti-*Mycobacterium tuberculosis* activity of spiropyrrolidine heterocyclic hybrids

The synthesized spirooxindolopyrrolidine embedded chromanones 5a–j were tested for their antimycobacterium activity against *Mtb* H37Rv, isoniazid-resistant (*katG* or *inhA* promoter mutation) clinical *Mtb* isolates using the Microplate Alamar Blue Assay (MABA) method and Middlebrook 7H9 broth + OADC (Growth supplement), with isoniazid used as the reference standard drug. Initially, we evaluated the first series of spirooxindolopyrrolidines 5a–j against *Mtb* H37Rv and *Mtb* isoniazid-resistant (*katG* or *inhA* promoter mutation). Among the compounds, four spirocompounds displayed potent antitubercular activity against the tubercular organisms tested (Table 2). Thus, the compound 5c that possessed 2-chloro substituted derivative showed excellent activity against H37Rv with MIC value 0.78 μg mL^−1^, which further showed potent activity against *inhA* promoter and *katG* mutation clinical isolates with MIC values 0.19 μg mL^−1^ and 0.39 μg mL^−1^, respectively. Similarly, the compound 5d bearing 4-chlorosubsitution on the aryl ring displayed good activity against H37Rv with MIC value 1.56 μg mL^−1^ and the compound showed excellent activity against *inhA* promoter and *katG* mutation isolates with MIC values 0.39 μg mL^−1^ and 1.56 μg mL^−1^, respectively. Compound 5h with 2-fluorosubstitution on the aryl ring showed significant activity against H37Rv with MIC value 1.56 μg mL^−1^, which displayed significant activity against *inhA* promoter and *katG* mutation isolates with MIC values 0.78 and 1.56 μg mL^−1^, respectively. Likewise, compound 5i bearing 4-fluoro on the phenyl ring exhibited potent activity against H37Rv with MIC value 0.39 μg mL^−1^, which showed 0.09 μg mL^−1^ and 0.19 μg mL^−1^ against *inhA* promoter and *katG* mutation isolates, respectively. The above mentioned antitubercular results revealed that the spirooxindolopyrrolidine integrated chromanones with halogen substituted derivative showed significant activity against the tubercular organisms tested. In particular, the compound possessing a fluoro substitution on the aryl ring showed more potent activity which was comparable against the standard drug isoniazid (INH).

**Table 2:** Antimycobacterium tubercular activity of spiropyrrolidines, 5a–j

Compounds	H37Rv (MIC μg mL^−1^)	*inhA* promoter mutation (MIC μg mL^−1^)	*katG* mutation (MIC μg mL^−1^)
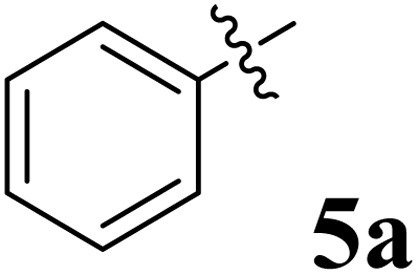	>50	>50	>50
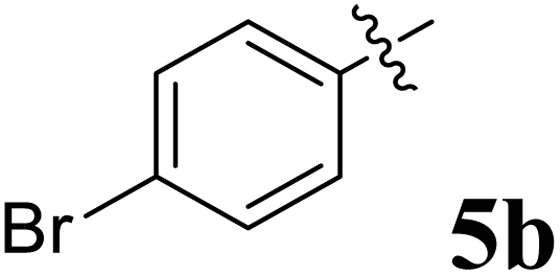	>50	>50	>50
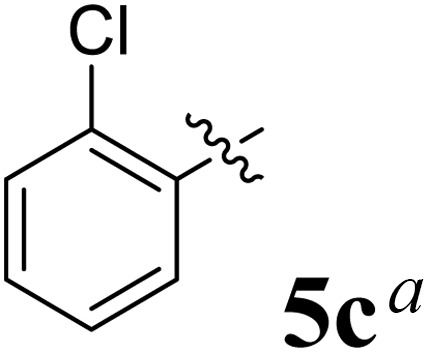	**0.78**	**0.19**	**0.39**
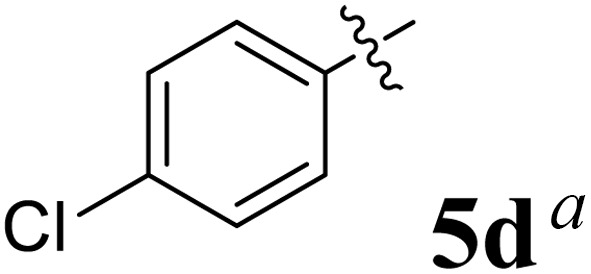	**1.56**	**0.39**	**1.56**
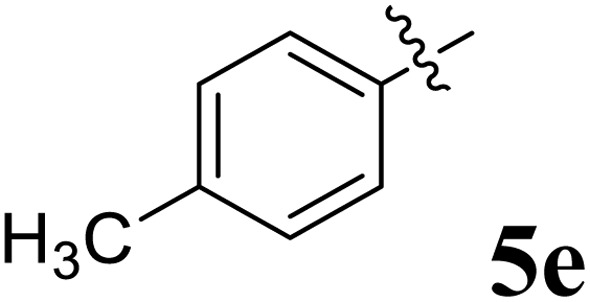	>50	>50	>50
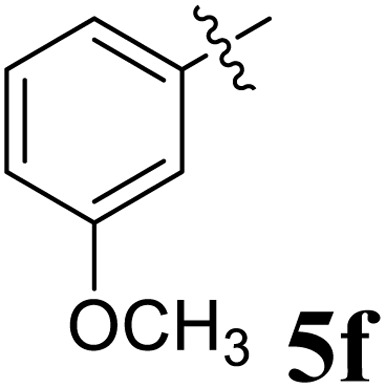	>50	>50	>50
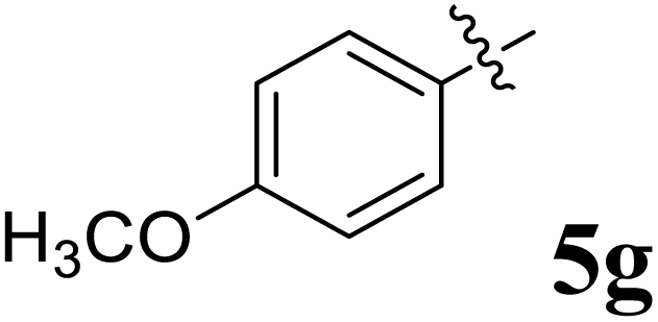	>50	>50	>50
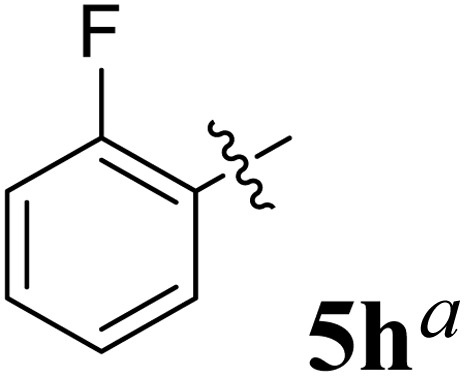	**1.56**	**0.78**	**1.56**
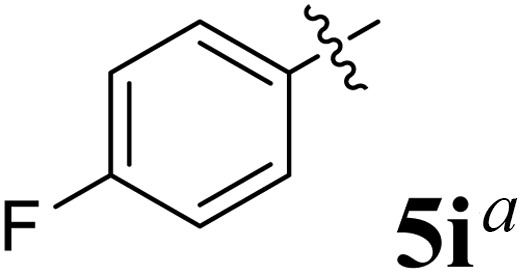	**0.39**	**0.09**	**0.19**
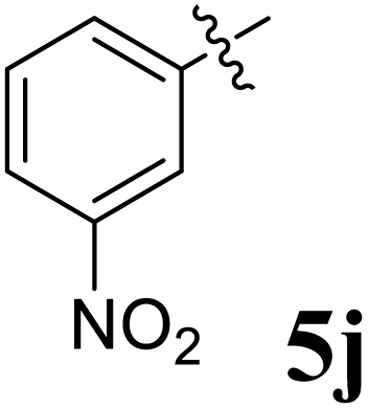	>50	>50	>50
Isoniacid (INH)	0.19 μM	1.56 μM	12.5 μM

a Compounds with promising activity.

### 2.3. Molecular docking study

Molecular docking studies showed that the ligand had a strong binding affinity with the active sites of Type II dehydroquinase (−9.59) and decaprenyl phosphoryl-beta-d-ribose oxidase from *Mycobacterium tuberculosis* (−10.81). Further, it showed a stronger binding affinity compared to known natural ligands. Hydrophobic interactions were formed between the amino acids PRO11, ASN12, LEU13, LEU16, TYR24, ASN75, GLY77, GLY78 of Type II dehydroquinase (1H0R) active sites and the ligand. In the case of decaprenyl phosphoryl-beta-d-ribose oxidase, interactions with both the hydrogen bond (CYS129) and hydrophobic amino acids (ILE131, ALA417, ARG58, GLY125, VAL121, SER59, THR118, TYR60 and TYR415) were observed. This could probably contribute to stronger binding affinity of the ligand with the decaprenyl phosphoryl-beta-d-ribose oxidase compared to Type II dehydroquinase.

### 2.4. ADME studies

Absorption, distribution, metabolism, and excretion (ADME) properties of the ligand were assessed using lipophilicity, pharmacokinetics, water solubility, physio-chemical properties and drug-likeness. The ligand lacked flexibility and polar interactions. This was reflected in the interactions observed in the docking studies. However, in general, the ligand exhibited good druggability properties. Swiss Target Prediction was used to bioinformatically forecast additional possible targets to understand the cross-reactivity of the drug molecule. Swiss Target Prediction uses molecular similarity and Shape to forecast prospective targets. Our study demonstrated that the ligand has a 46.7% chance of binding to proteases, 26.7% with G-protein coupled receptors and 13.3% with hydrolases. Overall, the molecule shows good properties in order to be used as a possible drug for TB.

#### 2.4.1 ADME studies


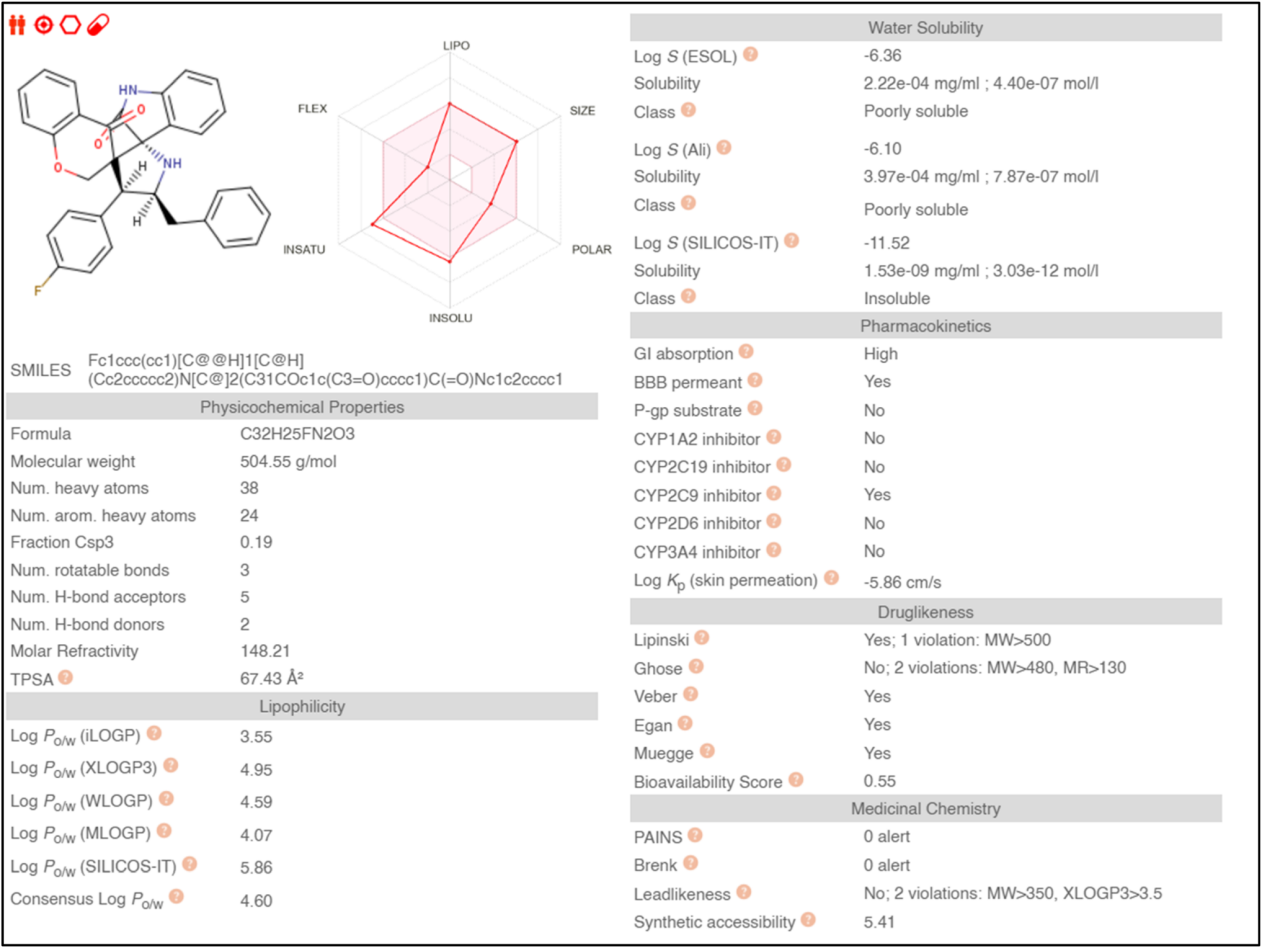


#### 2.4.2 Type II dehydroquinase from *Mycobacterium tuberculosis* (1H0R)


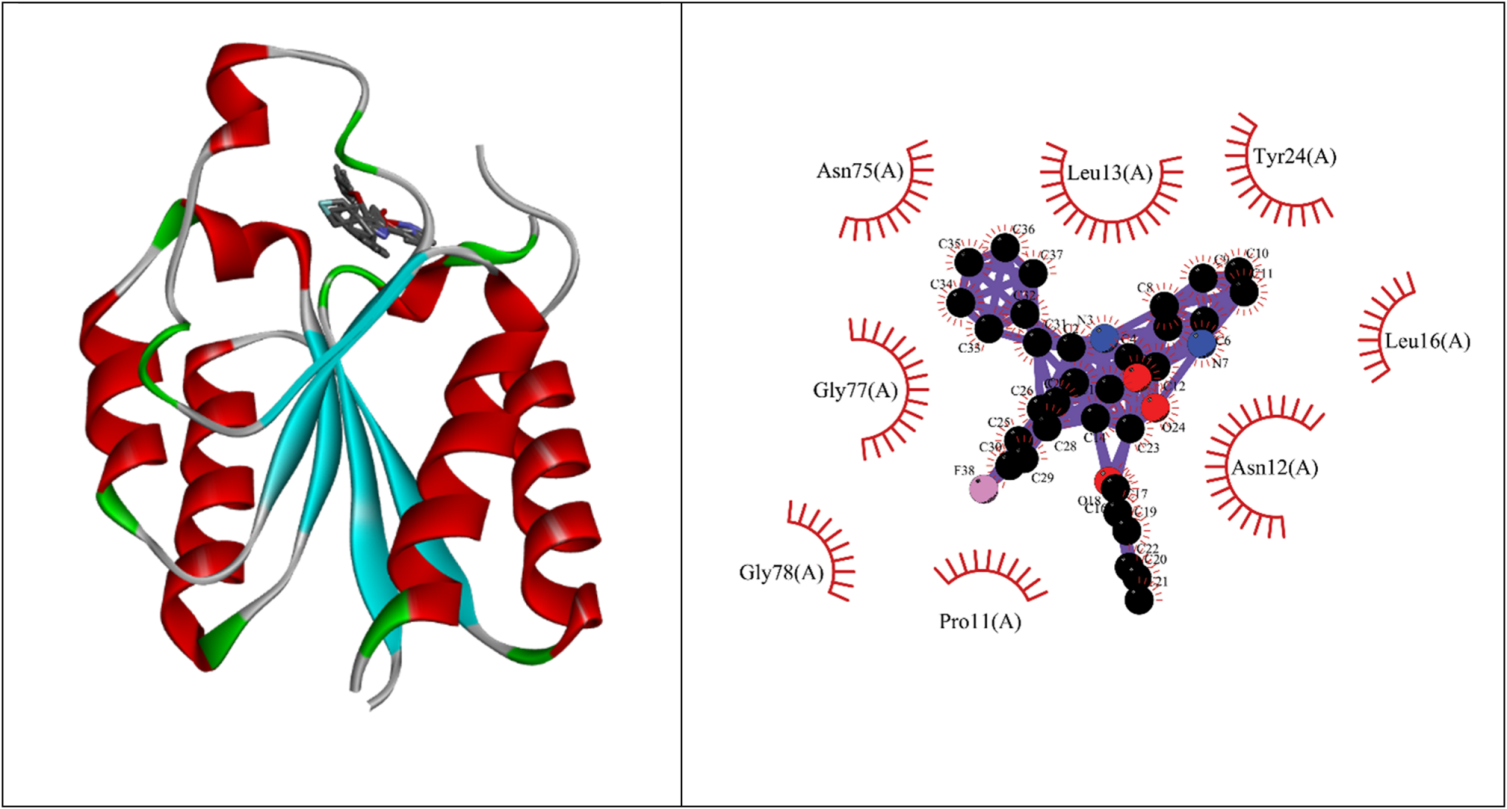
Binding energy of 5i with the active site of 1H0R (decaprenyl phosphoryl-beta-d-ribose oxidase from *Mtb*): −10.81.

Binding energy of known ligand (FAD) with the active site of 1H0R (decaprenyl phosphoryl-beta-d-ribose oxidase from *Mtb*): −7.05.

#### 2.4.3 Decaprenyl phosphoryl-beta-d-ribose oxidase from Mtb (4FDN)


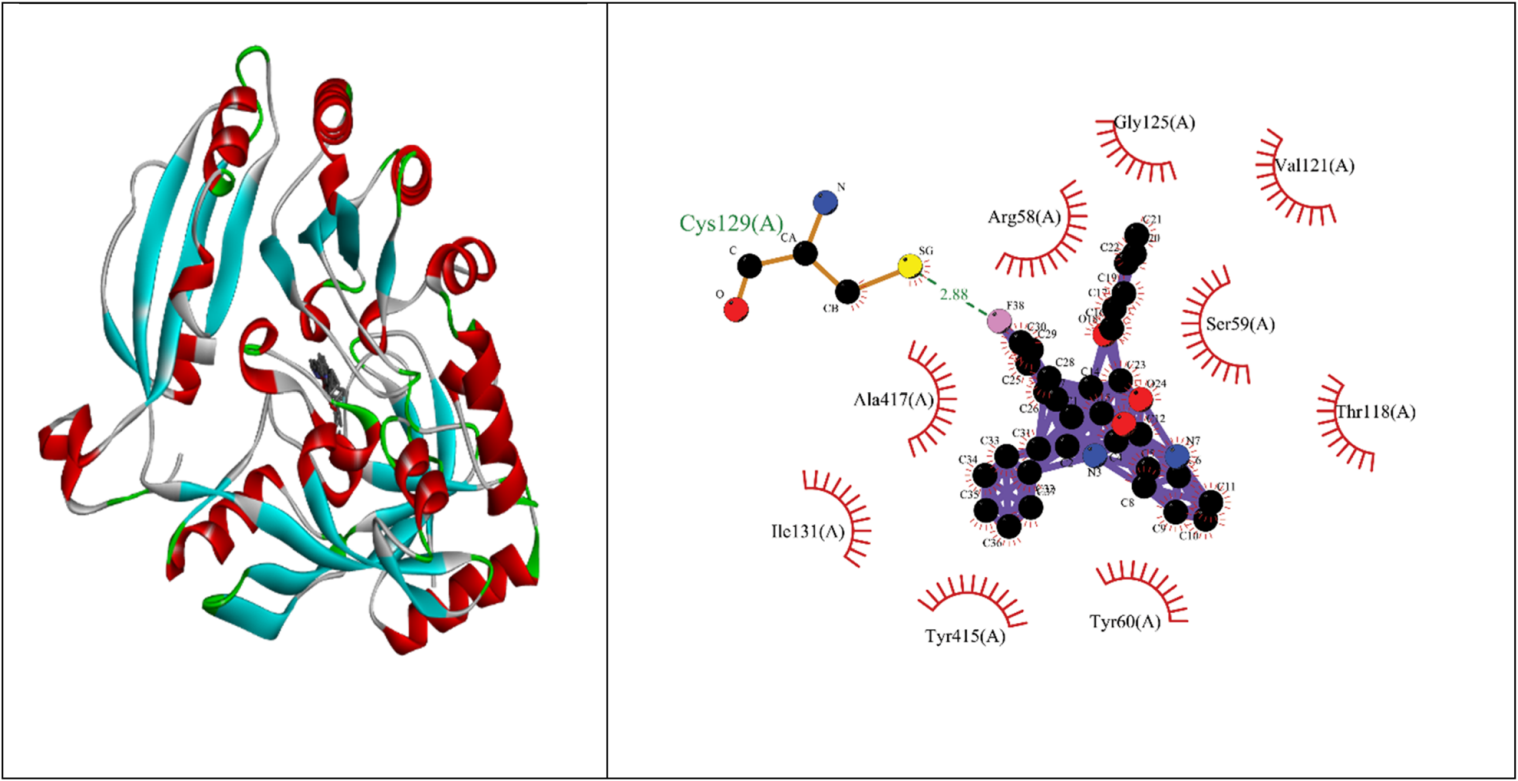
Binding energy of 5i with the active site of 1H0R (decaprenyl phosphoryl-beta-d-ribose oxidase from *Mtb*): −10.81.

Binding energy of known ligand (FAD) with the active site of 1H0R (decaprenyl phosphoryl-beta-d-ribose oxidase from *Mtb*): −7.05.

### 2.5. Swiss target prediction


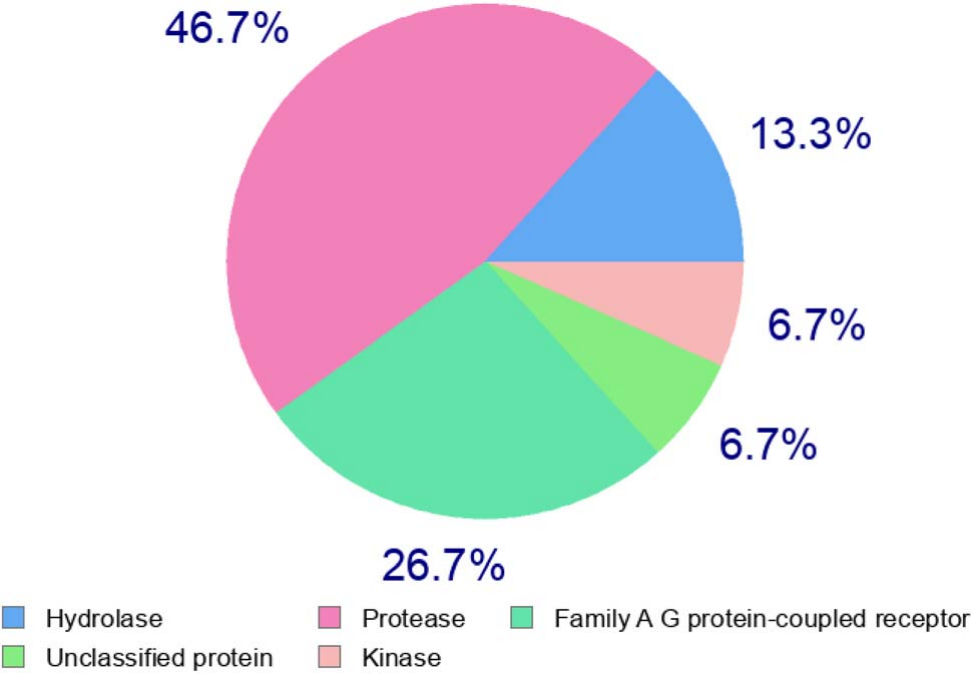


**Table:** 

Target	Common name	Target class	Probability
Acetylcholinesterase	ACHE	Hydrolase	0.121906255
Thrombin	F2	Protease	0.121906255
Urotensin II receptor	UTS2R	Family A G protein-coupled receptor	0.121906255
ADAMTS5	ADAMTS5	Protease	0.121906255
Phospholipase D1	PLD1	Hydrolase	0.121906255
Dipeptidyl peptidase II	DPP7	Protease	0.121906255
Dipeptidyl peptidase VIII	DPP8	Protease	0.121906255
Dipeptidyl peptidase IX	DPP9	Protease	0.121906255
Menin	MEN1	Unclassified protein	0.121906255
Gonadotropin-releasing hormone receptor	GNRHR	Family A G protein-coupled receptor	0.121906255

#### 2.5.1 Boiled egg representation


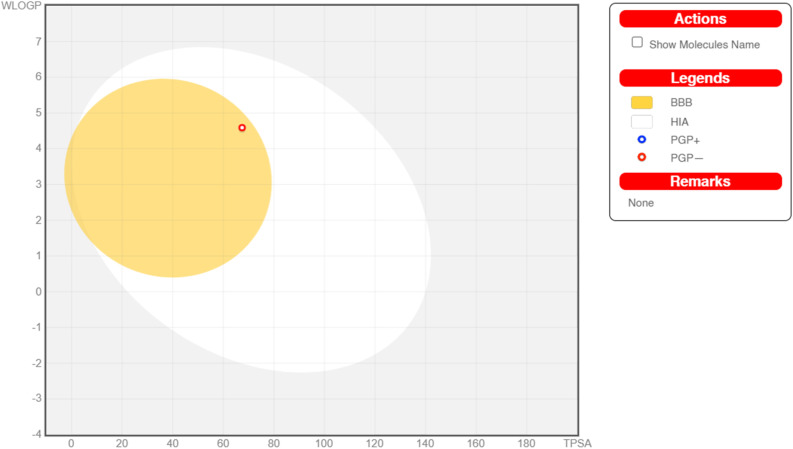


## 3. Conclusion

A series of structurally new class of spirooxindolopyrrolidine tethered chromanones were synthesized in quantitative yields using cycloaddition methodology. The spiro compounds were evaluated for their antitubercular activity against *Mycobacterium tuberculosis* H37Rv, isoniazid-resistant (*katG* and *inhA* promoter mutation) clinical *Mycobacterium tuberculosis* isolates. The spirooxindolopyrrolidine integrated chromanones that possessed an electron withdrawing group substituted derivative displayed significant activity against tested *Mycobacterium tuberculosis* H37Rv, as well as isoniazid-resistant *Mycobacterium tuberculosis* strains. Among them, the compound bearing fluorine derivative displayed significant activity against tested antimycobacterial pathogens which were comparable to the activity of the reference standard drug isoniazid. Molecular docking simulation was also performed with the most active compounds, and the docking results in relation with ADME and receptor specificity studies confirmed the possible role of the molecule as a potential drug for treating TB efficiently.

## 4. Experimental section

### 4.1. Preparation of spiropyrrolidines, 5a–j

The reaction consisted of a mixture of l-phenylalanine 2 (1.1 mmol), diketone 1 and dipolarophile 4a–j (1 mmol) was heated to reflux in MeOH at 75 °C for 2 h. TLC was used to verify the completeness of the reaction, and filtration was used to obtain the pure products.

Compound 5c: ^1^H NMR: *δ*_H_ 2.68–2.72 (1H, dd, *J* = 13.5, 5.0 Hz), 2.88–2.92 (1H, m), 3.28 (1H, d, *J* = 12.0 Hz), 4.34 (1H, *J* = 12.0 Hz), 4.64–4.68 (1H, m), 4.73 (1H, d, *J* = 9.5 Hz), 6.44 (1H, ArH, t, *J* = 8.0 Hz), 6.49 (1H, ArH, d, *J* = 8.0 Hz), 6.54 (1H, ArH, d, *J* = 7.0 Hz), 6.71 (1H, ArH, d, *J* = 7.5 Hz), 6.80–6.84 (2H, ArH, m), 6.99 (1H, ArH, t, *J* = 7.5 Hz), 7.07–7.16 (6H, ArH, m), 7.27 (2H, ArH, t, *J* = 8.0 Hz), 7.71–7.73 (1H, ArH, dd, *J* = 8.0, 1.5 Hz), 7.88 (1H, ArH, d, *J* = 8.0 Hz), 10.52 (1H, s, 1H); ^13^C NMR: *δ*_C_ 39.7, 50.5, 59.8, 63.9, 72.4, 72.7, 109.5, 117.1, 121.1, 121.3, 121.9, 126.2, 126.3, 127.0, 127.6, 127.9, 128.4, 129.1, 129.3, 129.7, 131.1, 135.3, 135.4, 135.9, 139.6, 142.5, 161.4, 178.7, 192.1; mass *m*/*z*: 521 (M^+^).

Compound 5d: ^1^H NMR: *δ*_H_ 2.75 (2H, d, *J* = 6.0 Hz), 3.29 (1H, d, *J* = 12.0 Hz), 4.25 (1H, d, *J* = 11.0 Hz), 4.43–4.48 (1H, m), 4.73 (1H, d, *J* = 12.0 Hz), 6.40 (1H, ArH, d, *J* = 8.0 Hz), 6.51–6.57 (2H, m, ArH), 6.68–6.73 (2H, m, ArH), 6.80 (1H, t, *J* = 8.0 Hz, ArH), 7.06–7.17 (7H, m, ArH), 7.31–7.35 (ArH, m, 3H), 7.41 (1H, ArH, d, *J* = 8.0 Hz), 10.34 (1H, s, NH); ^13^C NMR: *δ*_C_ 39.8, 52.9, 61.1, 62.3, 70.6, 71.8, 109.2, 117.0, 120.9, 121.1, 121.4, 126.2, 127.6, 127.7, 128.1, 128.4, 129.1, 129.2, 129.5, 132.4, 135.8, 139.7, 142.5, 161.0, 179.5, 191.9; mass *m*/*z*: 521 (M^+^).

Compound: 5h: ^1^H NMR: *δ*_H_ 2.84–2.88 (2H, m), 3.27–3.40 (1H, m), 4.60–4.68 (3H, m), 6.51–6.57 (3H, m, ArH), 6.72–6.83 (3H, m, ArH), 7.03–7.34 (9H, m, ArH), 7.63–7.68 (2H, m, ArH), 10.48 (1H, s, NH); ^13^C NMR: *δ*_C_ 40.0, 59.9, 62.6, 67.9, 72.0, 72.3, 109.4, 117.1, 118.5, 121.1, 121.7, 121.9, 122.6, 124.9, 126.3, 127.4, 127.9, 128.5, 129.3, 129.4, 129.9, 135.9, 136.9, 139.7, 142.6, 160.5, 161.4, 162.5, 178.9, 191.9; mass *m*/*z*: 504 (M^+^).

Compound 5i: ^1^H NMR: *δ*_H_ 2.77–2.86 (2H, m), 3.18 (1H, d, *J* = 8.8 Hz), 4.29 (1H, d, *J* = 10.0 Hz), 4.40–4.51 (1H, m), 4.76 (1H, d, *J* = 11.6 Hz), 6.43 (1H, d, *J* = 7.2 Hz, ArH), 6.57–6.62 (2H, m, ArH), 6.74–6.77 (2H, m, ArH), 6.80–6.88 (1H, m, ArH), 7.11–7.21 (8H, m, ArH), 7.44 (3H, d, *J* = 8.0 Hz, ArH), 10.34 (1H, s, NH); ^13^C NMR: *δ*_H_ 39.3, 52.4, 54.1, 60.6, 64.9, 70.1, 108.6, 115.6, 116.4, 120.5, 120.6, 125.9, 127.7, 127.8, 128.1, 128.6, 128.9, 129.1, 132.4, 135.2, 135.5, 139.3, 141.9, 160.1, 160.5, 162.5, 179.0, 191.6; mass *m*/*z*: 504 (M^+^).

### 4.2. Anti-tubercular screening

The strains H37Rv of *Mycobacterium tuberculosis*, clinical isolates with *inhA* promoter mutation and *katG* mutation was streaked onto 7H10+OADC agar plates and incubated at 37 °C. Pure colonies grown on OADC-enriched liquid 7H9 medium were grown to mid-log phase. A cultural growth was followed by inoculation into 7H9 medium on 96-well plates at progressively higher concentrations of the testing chemicals, using approximately 4 × 10^5^ CFU mL^−1^ in 200 μL of culture per well. Plates were incubated for one week at 37 °C before receiving 32.5 μL of a Resazurin-tween mixture (8 : 5 ratio of 0.6 mM Resazurin in PBS to 20% Tween 80). Resorufin produced by fluorescent resorufin is used for determining the minimum inhibitory concentration (MIC) of compounds.^31^

### 4.3. Methodology

Molecular docking studies were performed using Autodock (MGL-Tools) and Cygwin. Type II dehydroquinase (1H0R) and decaprenyl phosphoryl-beta-d-ribose oxidase from *Mtb* (4FDN) were used as target proteins. The methodology and the active sites followed for docking studies were obtained from literature.^32,33^ Docking studies were also carried out with the natural ligands and the binding efficiency of the ligands were assessed. The ligand interaction plots were made using Discovery Studio and LigPlus softwares. Subsequently, ADME properties (absorption, distribution, metabolism and excretion) and other potential targets for the ligand was assessed using Swiss ADME online portals (http://www.swissadme.ch/) and Swiss Target Prediction (https://www.swisstargetprediction.ch/). The interaction image was made using Liplot.^34^

## Conflicts of interest

There are no conflicts to declare.

## Supplementary Material

RA-014-D4RA01501K-s001

RA-014-D4RA01501K-s002
