# Supplementary material for: Activity against Mycobacterium tuberculosis of a new class of spirooxindolopyrrolidine embedded chromanone hybrid heterocycles
Source: RSC Adv. 2024 Apr 11;14(17):11604–13. doi: 10.1039/d4ra01501k (PMC11008671; doi:10.1039/d4ra01501k)
Supplement: RA-014-D4RA01501K-s001 [file RA-014-D4RA01501K-s001.pdf]

Electronic Supplementary Material (ESI) for RSC Advances.
This journal is © The Royal Society of Chemistry 2024

Supplementary Information

Activity against Mycobacterium tuberculosis of a new class of spirooxindolopyrrolidine embedded chromanone hybrid heterocycles

Manal Fahad Alkaltham,[a] Abdulrahman I. Almansour,[a] Natarajan Arumugam,[a]* Siva Krishna Vagolu[b,c]*, Tone Tønjum [b,c] Shatha Ibrahim Alaqeel,[d] Sai Swaroop Rajaratnam,[e] Venkatesh Sivaramakrishnan,[e]

[a] Department of Chemistry, College of Science, King Saud University, P.O. Box 2455, Riyadh 11451, Saudi Arabia

[b] Department of Microbiology, University of Oslo, N-0316 Oslo, Norway

[c] Department of Microbiology, Oslo University Hospital, N-0424 Oslo, Norway

[d]Department of Chemistry, College of Science, King Saud University (034), Riyadh 11495, Saudi Arabia

[e]Disease Biology Lab, Department of Biosciences, Sri Sathya Sai Institute of Higher Learning, Prasanthi Nilayam, Anantapur, Andhra Pradesh, India

---

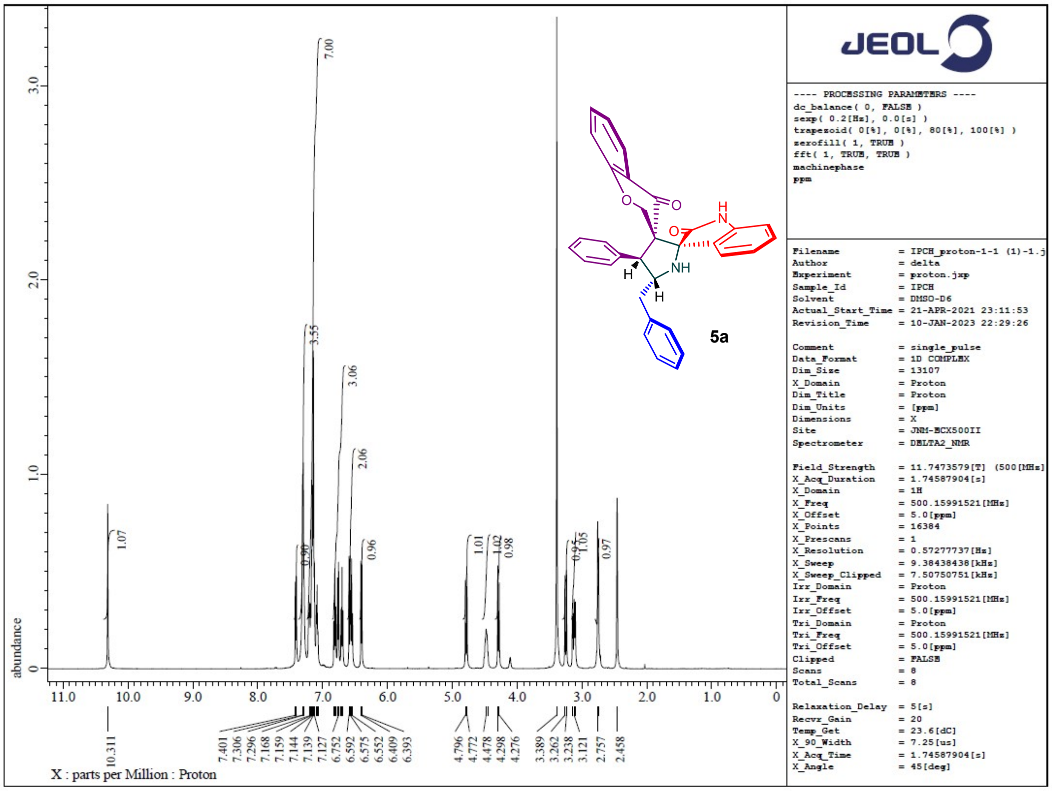

Figure S1. ^{1}H NMR spectrum of **5a**

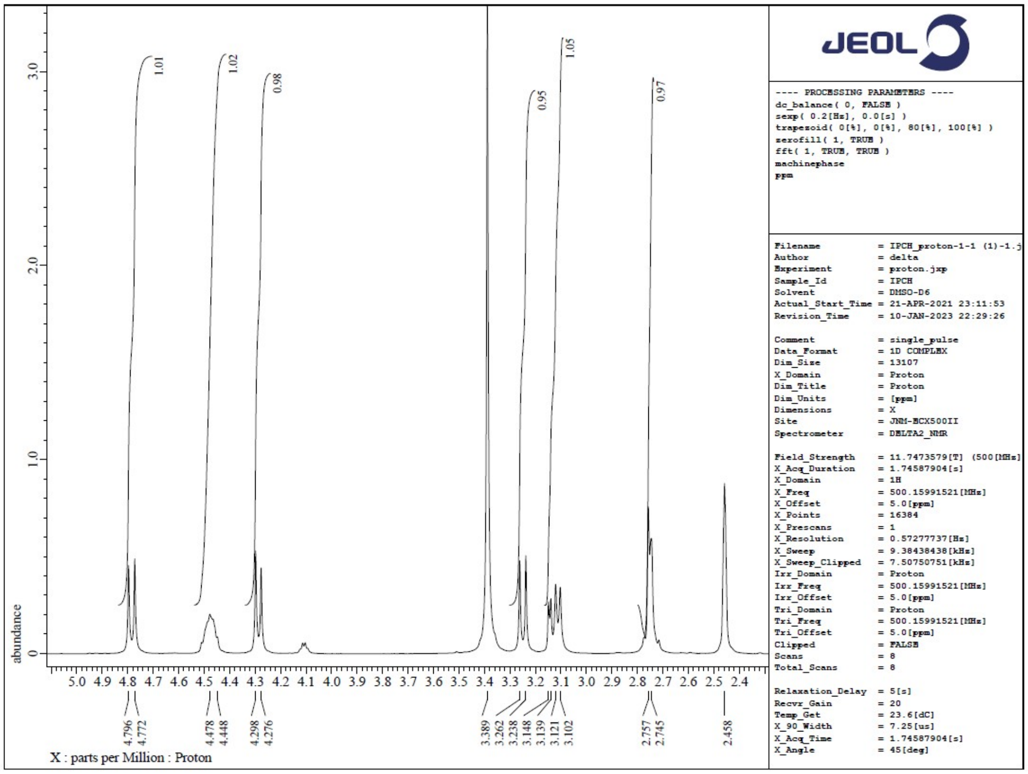

Figure S2. Expanded ^{1}H NMR spectrum of **5a**

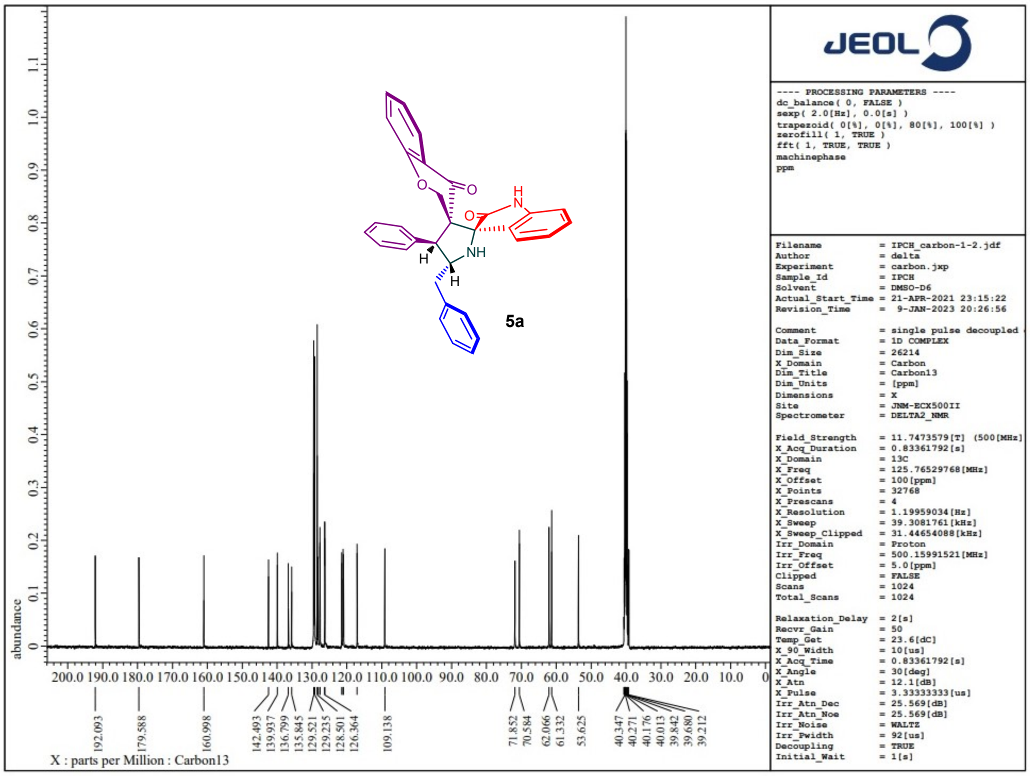

Figure S3. ^{13}C NMR spectrum of **5a**

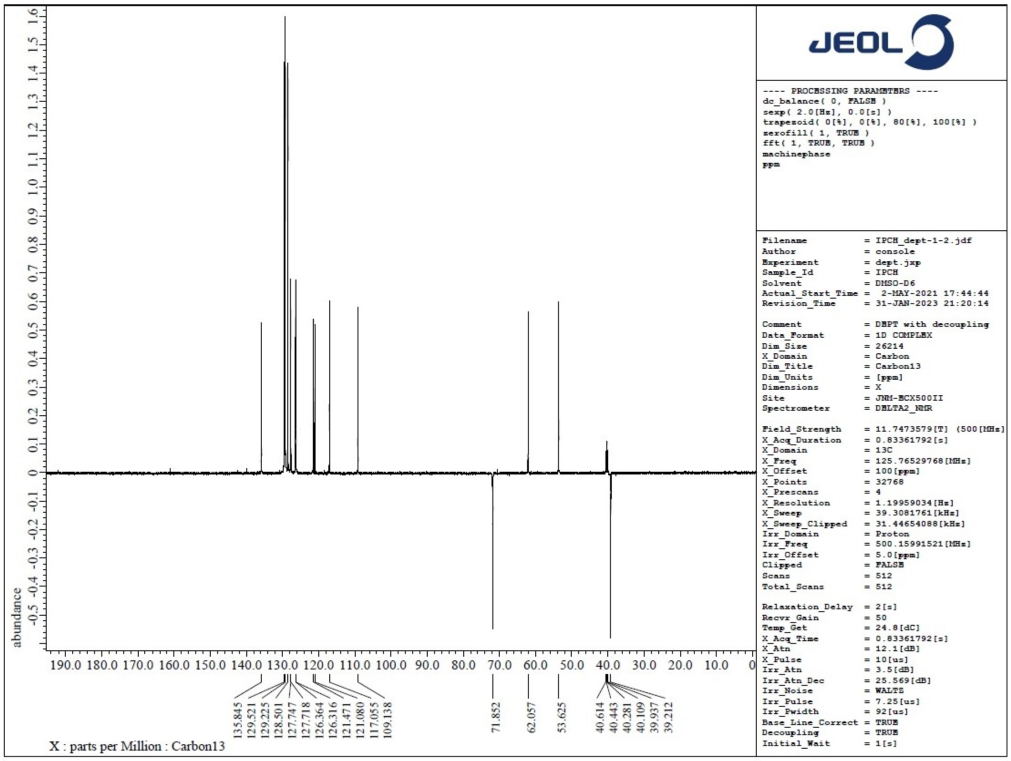

Figure S4. DEPT-135 spectrum of **5a**

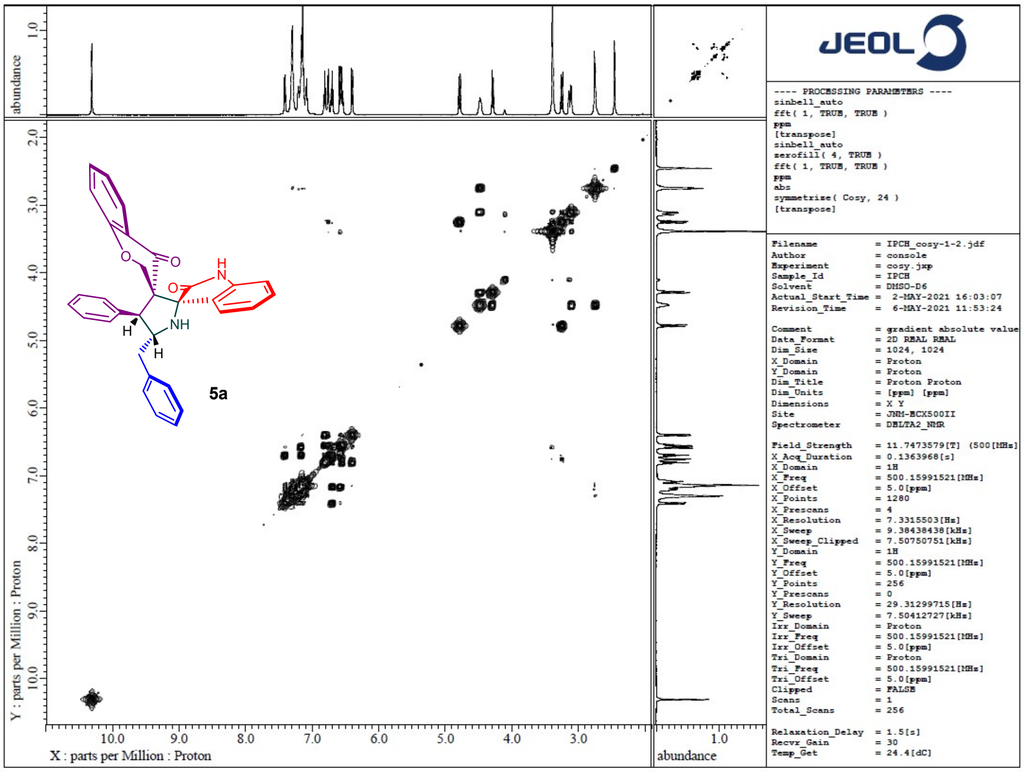

Figure S5. $^{1}H,^{1}H$-COSY spectrum of **5a**

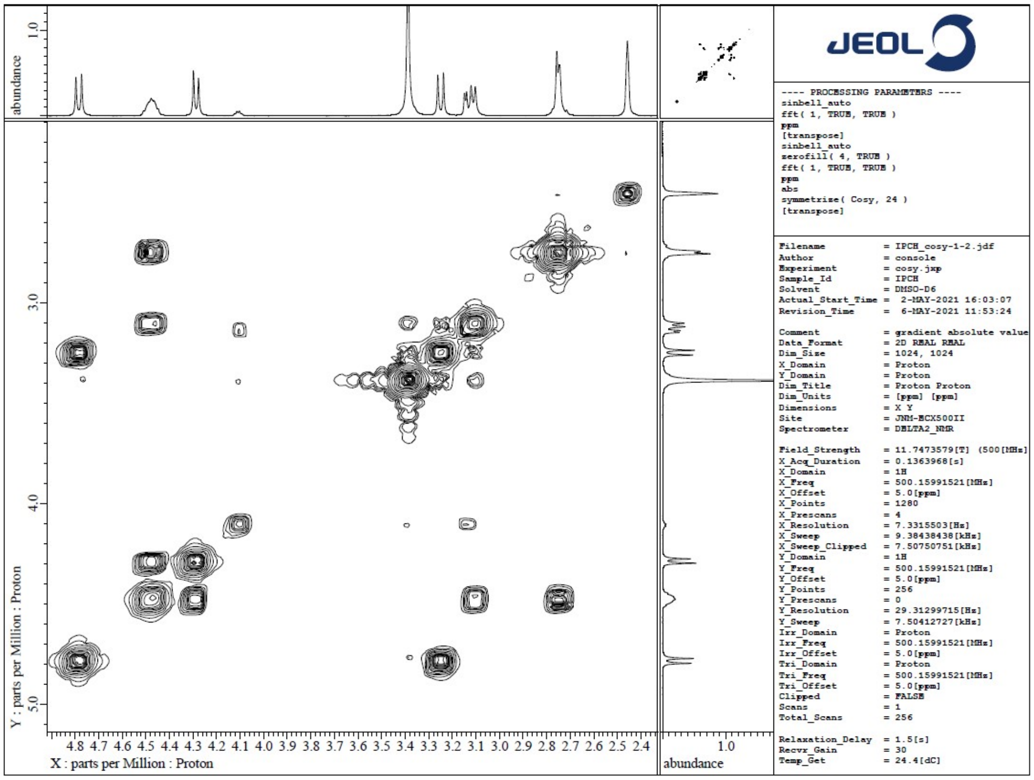

Figure S6. Expanded $^{1}H,^{1}H$-COSY Expansion spectrum of **5a**

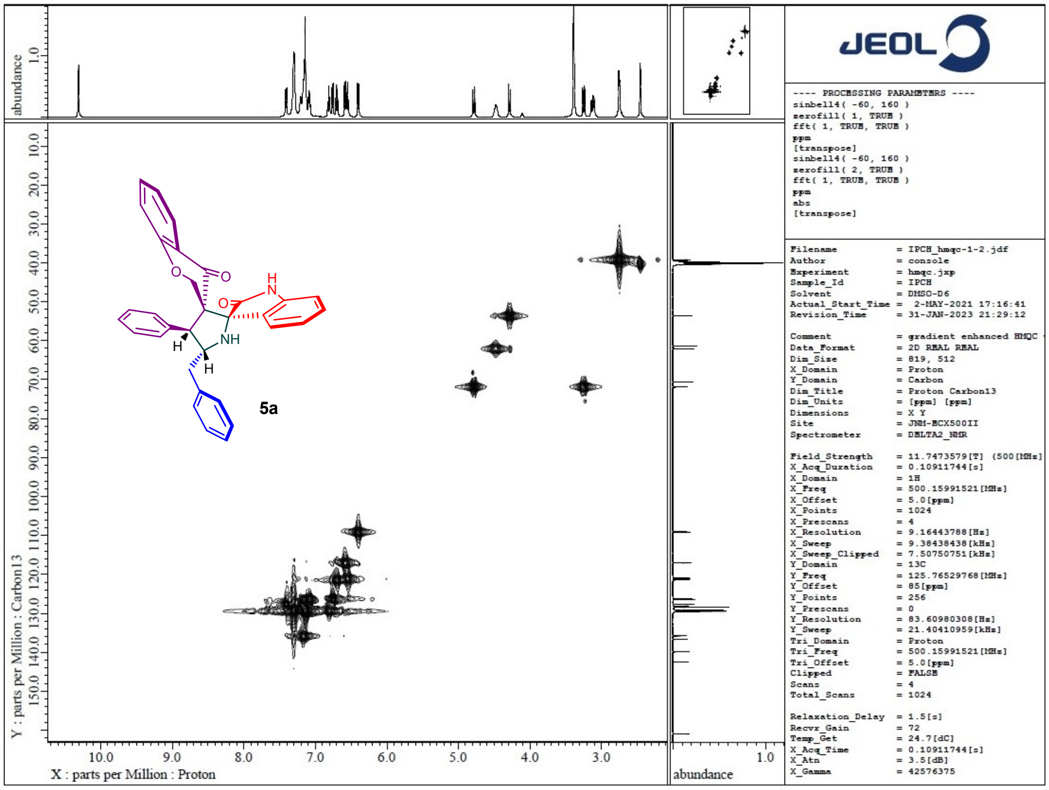

Figure S7. ^{13}C, H-COSY (HMQC) Expansion spectrum of **5a**

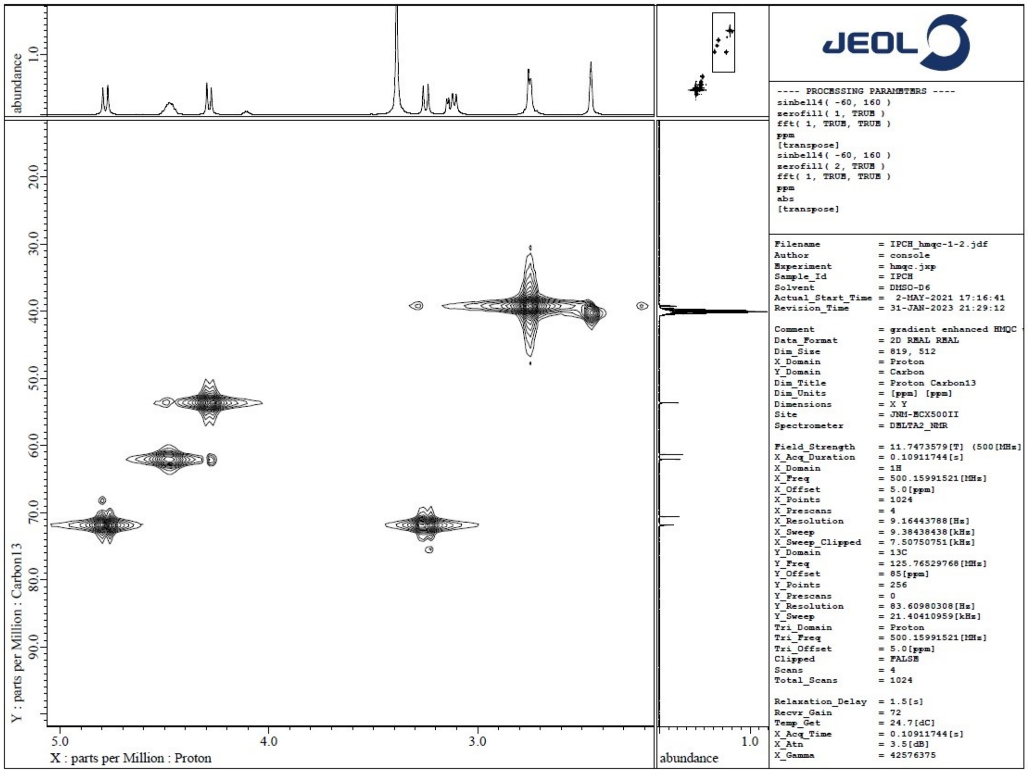

Figure S8. Expanded ^{13}C, H-COSY (HMQC) Expansion spectrum of **5a**

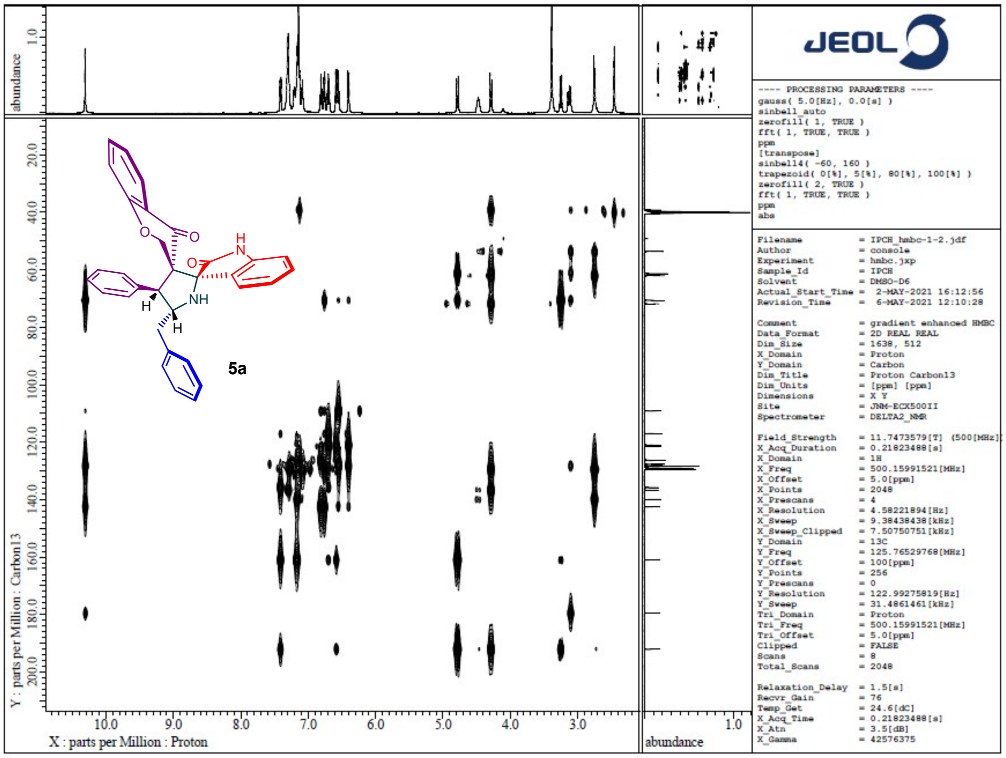

Figure S9. HMBC spectrum of **5a**

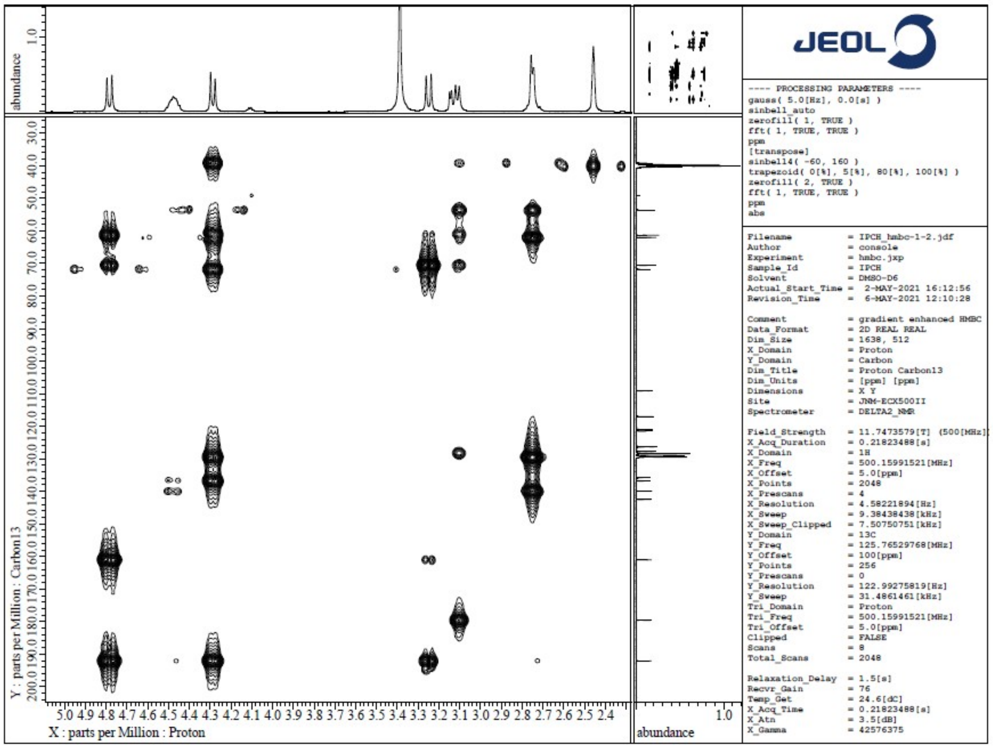

Figure S10. Expanded HMBC spectrum of **5a**

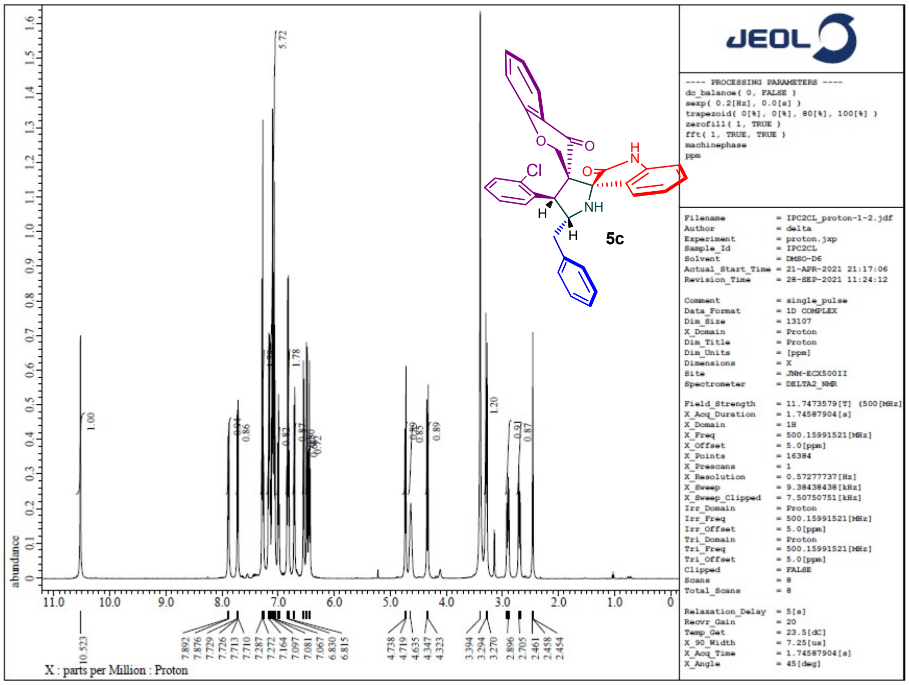

Figure S11. ^{1}H NMR spectrum of **5c**

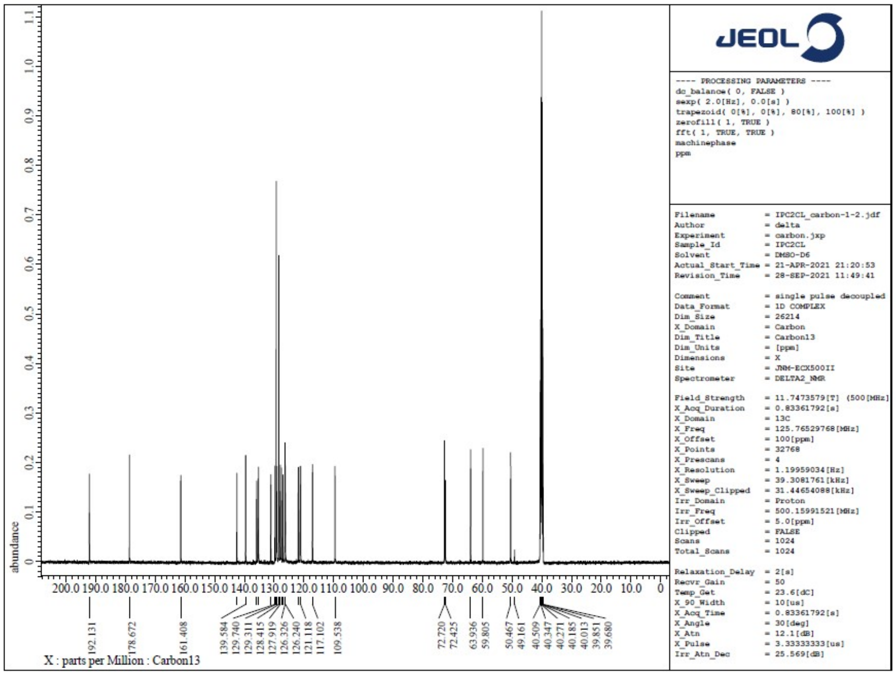

Figure S12. ^{1}H NMR spectrum of **5c**

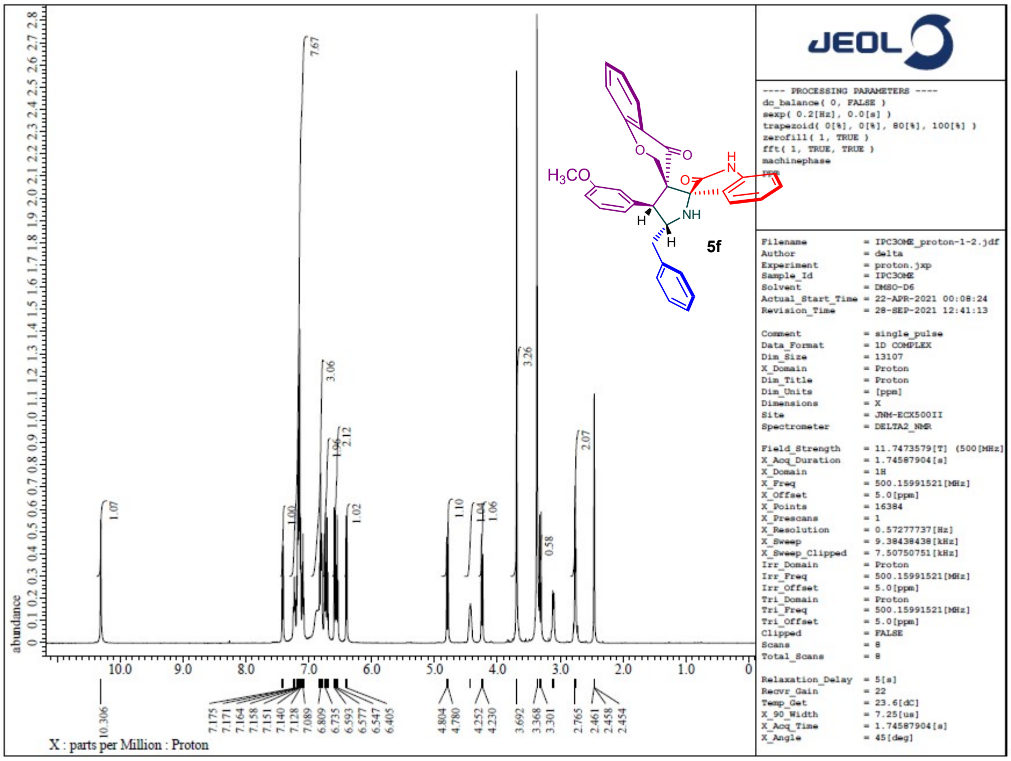

Figure S13. ^{1}H NMR spectrum of **5f**

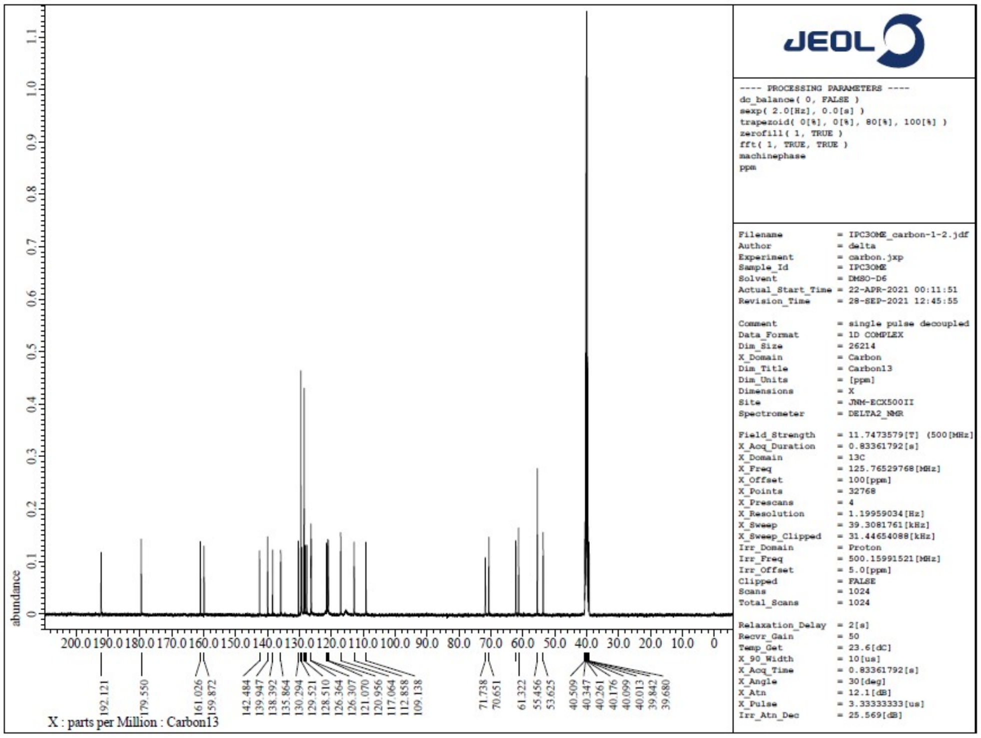

Figure S13. ^{1}H NMR spectrum of **5f**

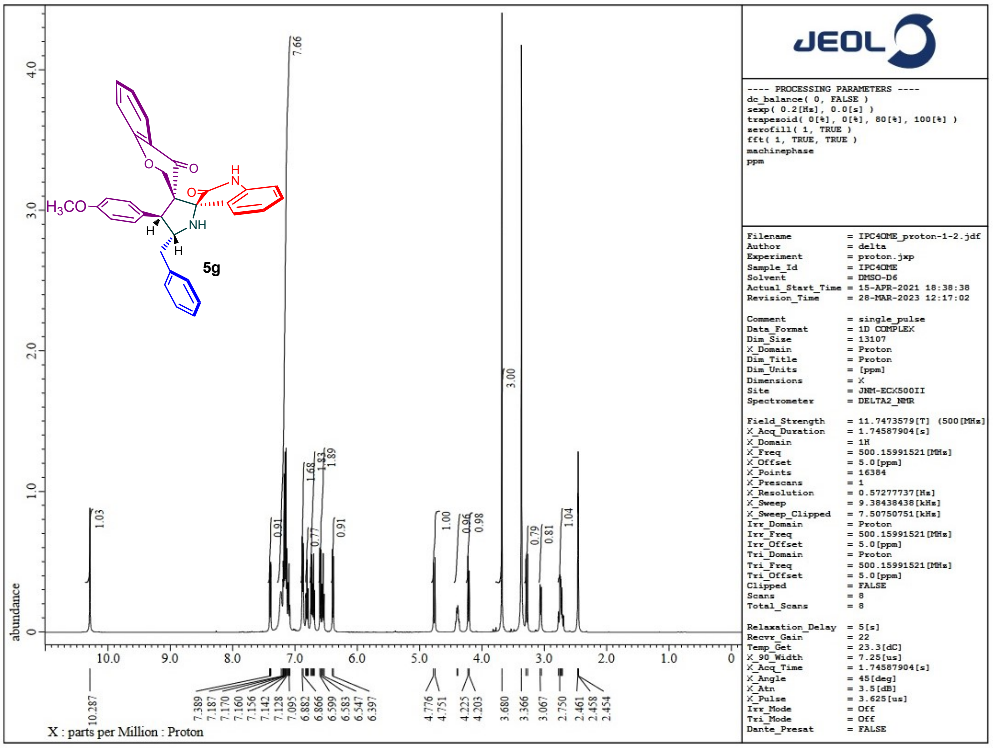

Figure S11. ^{1}H NMR spectrum of **5g**

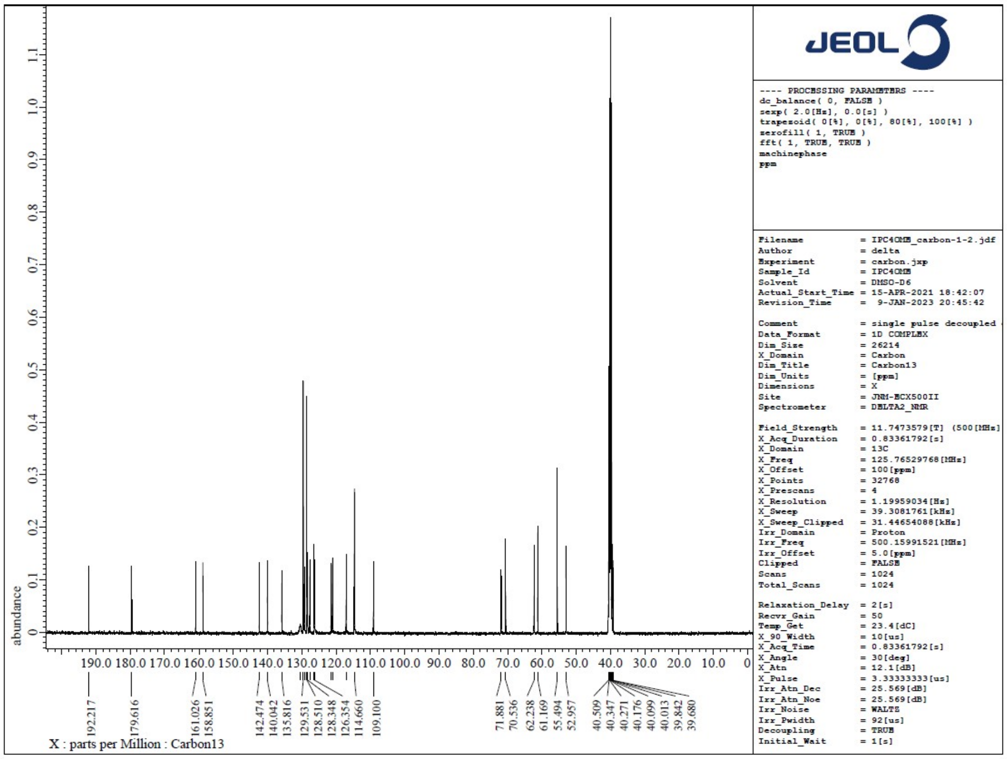

Figure S12. ^{13}C NMR spectrum of **5g**

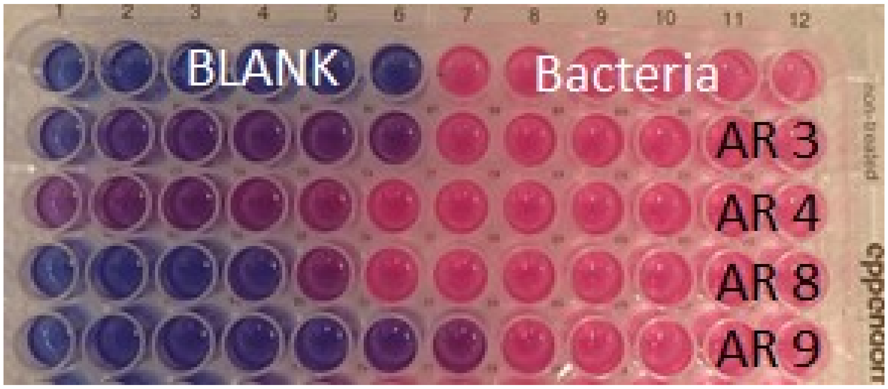

Figure S13: Culture media plates of Mycobacterium tuberculosis for the Anti-Mycobacterium tuberculosis activity of spiropyrrolidine heterocyclic hybrids **(**AR3-9 represent as compound **5c**, **5d**, **5h** and **5i**)

Spectral discussion

The structure of compounds agreed with their 1D and 2D NMR spectroscopic analysis as evidenced for a representative compound **5a**. The IR spectrum of **5a** showed peaks at 1717 and 1687 cm^{-1} belonging to oxindole and chromanone carbonyl stretching frequency. In the ^{1}H NMR spectrum of **5a**, the doublet at δ 4.29 (J = 11.0 Hz) ppm was assigned to benzylic hydrogen (H-4) of the pyrrolidine ring which showed (i) H, H-COSY correlation with the multiplets at δ 4.44-4.48 ppm assignable H-5 hydrogen of pyrrolidine ring (ii) HMBCs with C-3 C-5, C-6 and C-7 at 61.3, 62.1, 39.2 and 71.9 ppm, respectively, besides showing a correlation with chromanone carbonyl at δ 192.1 ppm. H-5 hydrogen displayed correlation with the multiplets at δ 2.75-2.76 ppm assignable to H-6 hydrogens which showed (i) HMBCs with C-4 and C-5 at 53.6 and 62.1 ppm (ii) H, H-COSY correlations to each other hydrogens. The chromanone methylene hydrogens (C-7) showed two doublets at δ 3.43 and 4.78 (J =12.0 Hz) ppm. H-7 hydrogens showed HMBCs with spiro carbon (C-2) and chromanone carbonyl carbon (C-4′) at δ 70.6 and 192.1 ppm. Further, the methine and methylene carbon signal was assigned through DEPT-135 spectrum. The two spiro carbons were undoubtedly assigned through the absence spirocarbon signal in DEPT-135 at δ 70.6

(C-2) and 61.3 (C-3) ppm. The carbonyl signal was unambiguously assigned through HMQC spectroscopic analysis.

Compound, 5a: Obtained as white solid (89 %): IR (KBr): 1687, 1717, 3244, 3395, 3030, 1195 cm^{-1}; ^{1}H NMR (DMSO-d_6, 500 MHz): δ/ppm 2.75 (d, J = 6.0 Hz, 1H), 3.10-3.15 (m, 1H), 3.25 (d, J = 12.0 Hz, 1H), 4.29 (d, J = 11.0 Hz, 1H), 4.44-4.48 (m, 1H), 4,78 (d, J = 12.0 Hz, 1H), 6.40 (d, J = 8.0 Hz, 1H, ArH), 6.54-6.59 (m, 2H, ArH), 6.70 (t, J = 7.5 Hz, 1H, ArH), 6.76 (d, J = 7.5 Hz, 1H, ArH), 6.81 (t, J = 7.5 Hz, 1H, ArH), 7.08-7.12 (m, 3H, ArH), 7.13-7.20 (m, 7H, ArH), 7.30-7.31 (m, 1H, ArH), 7.41 (d, J = 8.0 Hz, 1H, ArH), 10.31 (s, 1H, NH); ^{13}C NMR (DMSO-d_6, 125 MHz): δ/ppm 39.2, 53.6, 61.3, 62.1, 70.6, 71.9, 109.1, 117.1, 120.9, 121.1, 121.5, 126.3, 126.4, 127.7, 127.8, 128.5, 129.2, 129.5, 135.8, 136.8, 139.9, 142.5, 160.9, 179.6, 192.1. Mass *m/z*: 487 (M^+)

Compound 5b: Obtained as white solid (94%): ^{1}H NMR (DMSO-d_6, 500 MHz): δ/ppm 2.74-2.75 (m, 1H), 3.20-3.30 (m, 2H) , 4.24 (d, J = 10.0 Hz, 1H), 4.42-4.43 (m, 1H), 4.73 (d, J = 12.0 Hz, 1H), 6.41 (d, J = 8.0 Hz, 1H, ArH), 6.53-6.60 (m, 2H, ArH), 6.70-6.75 (m, 2H, ArH), 6.81 (t, J = 7.0 Hz, 1H, ArH), 7.08-7.24 (m, 7H, ArH), 7.40-7.47 (m, 3H, ArH), 7.62-7.67 (m, 1H, ArH), 10.46 (s, 1H, NH); ^{13}C NMR (DMSO-d_6, 125 MHz): δ/ppm 39.8, 53.0, 61.2, 62.3, 70.6, 71.7, 109.2, 117.1, 118.5, 120.9, 121.1, 121.5, 126.4, 127.7, 128.1, 128.5, 129.3, 129.6, 132.1, 132.3, 132.8, 135.9, 136.3, 139.8, 142.5, 161.0, 179.5, 191.9; Mass m/z: 565 (M^+)

Compound 5e: Obtained as white solid (87%): ^{1}H NMR (DMSO-d_6, 500 MHz): δ/ppm 2.22 (s. 3H), 2.71-2.73 (m, 2H), 3.26 (d, J = 12.0 Hz, 1H), 4.22 (d, J = 10.5 Hz, 1H), 4.41-4.46 (m, 1H), 4.76 (d, J = 12.0 Hz, 1H), 6.38 (d, J = 7.5 Hz, 1H, ArH), 6.53-6.59 (m, 2H, ArH), 6.69-6.74 (m, 2H, ArH), 6.80 (t, J = 8.0 Hz, 1H, ArH), 7.09-7.23 (m, 10H, ArH), 7.37-7.39 (m, 1H, ArH), 10.29 (s, 1H, NH); ^{13}C NMR (DMSO-d_6, 125 MHz): δ/ppm 21.2, 39.8, 53.3, 61.2, 62.1, 70.5, 71.8, 109.1, 117.1, 120.9, 121.0, 121.5, 126.3, 126.4, 127.7, 128.3, 128.5, 128.8, 129.2, 129.5, 129.9, 133.6, 135.8, 136.9, 140.0, 142.5, 160.9, 179.6, 192.1; Mass m/z: 500 (M^+)

Compound ***5f:*** White solid (85%):^{1}H NMR: δ_H 2.75-2.77 (m, 2H), 3.31 (1H, d, J = 12.0 Hz), 3.69 (3H, s), 4.24 (1H, d, J = 11.0 Hz), 4.43-4.46 (1H, m), 4.79 (1H, d, J = 12.0 Hz), 6.40 (1H, d, J = 7.5 Hz, ArH), 6.53-6.59 (2H, m, ArH), 6.69-6.75 (2H, m, ArH), 6.79-6.83 (3H, m, ArH), 7.08-7.23

(8H, m, ArH), 7.40-7.42 (1H, dd, J = 8.0, 1.0 Hz, ArH), 10.31 (1H, s, NH); ^{13}C NMR: δ/ppm 39.2, 53.6, 61.3, 62.2, 70.7, 71.7, 109.1, 112.9, 117.1, 120.9, 121.1, 121.5, 126.3, 126.4, 127.7, 128.2, 128.5, 129.2, 129.5, 130.3, 135.9, 138.4, 139.9, 142.5, 159.9, 161.0, 179.6, 192.1; Mass m/z: 516 (M^+)

Compound 5g: Obtained as white solid (88%):^{1}H NMR (DMSO-d_6, 500 MHz): δ/ppm 2.71-2.77 (m, 1H), 3.06 (d, J = 9.0 Hz, 1H), 3.28 (d, J = 12.5 Hz, 1H), 3.68, (s, 3H), 4.21 (d, J = 11.0 Hz, 1H), 4.39-4.40 (m, 1H), 4.76 (d, J = 12.5 Hz, 1H), 6.39 (d, J = 7.5 Hz, 1H, ArH), 6.53-6.60 (m, 2H, ArH), 6.69-6.74 (m, 2H, ArH), 6.79-6.81 (m, 1H, ArH), 6.87 (d, J = 8.0 Hz, 2H, ArH), 7.08-7.22 (m, 8H, ArH), 7.39-7.41 (dd, J = 8.0, 2.0 Hz, 1H, ArH), 10.29 (s, 1H, NH); ^{13}C NMR (DMSO-d_6, 125 MHz): δ/ppm 39.7, 52.9, 55.5, 61.2, 70.5, 71.9, 109.1, 114.7, 117.1, 120.9, 121.1, 121.4, 126.3, 126.4, 127.7, 128.3, 128.4, 128.5, 129.5, 135.8, 140.0, 142.5, 158.9, 161.0, 179.6, 192.2; Mass m/z: 516 (M^+)

Compound 5j: Obtained as white solid (91%):^{1}H NMR (DMSO-d_6, 400 MHz): δ/ppm 2.75-2.79 (m, 1H), 2.88-2.90 (m, 1H), 4.40 (d, J = 10.0 Hz, 1H), 4.52-4.54 (m, 1H), 4.68 (d, J = 11.6 Hz, 1H), 5.41-5.42 (m, 1H), 6.46 (d, J = 8.0 Hz, 1H, ArH), 6.56-6.59 (m, 2H, ArH), 6.74-6.78 (m, 2H, ArH), 6.85 (t, J = 8.0 Hz, 1H, ArH), 7.08-7.13 (m, 3H, ArH), 7.21-7.24 (m, 1H, ArH), 7.47-7.57 (m, 2H, ArH), 7.74-7.87 (m, 2H, ArH), 8.03 (d, J = 6.8 Hz, 2H, ArH), 8.26-8.28 (m, 1H, ArH), 10.41 (s, 1H, NH); ^{13}C NMR (DMSO-d_6, 100 MHz): δ/ppm 39.5, 53.4, 61.3, 67.7, 70.7, 71.9, 109.4, 117.2, 118.6, 118.7, 121.1, 121.9, 122.7, 126.5, 128.0, 128.6, 129.8, 133.6, 134.8, 136.0, 136.1, 139.3, 139.5, 142.6, 148.4, 148.6, 161.2, 161.4, 179.6, 192.1; Mass m/z: 531 (M^+)
